# EUROCOVER-CLL: Reimbursement and accessibility of new treatments in relapsed/refractory chronic lymphocytic leukemia

**DOI:** 10.3389/fphar.2025.1629465

**Published:** 2025-09-02

**Authors:** Magdalena Monica, Sabina Becirovic, Ioana Bianchi-Manaila, Natasa Duborija-Kovacevic, Pero Draganic, Kristina Garouliene, Ariel Hammerman, Fanni Ispan, Boryana Ivanova, Agnes Männik, Gergo Meresz, Lusine Nazaryan, Oleksandra Oleshchuk, Alexandra Savova, Jana Skoupa, Nikola Stefanovic, Tomas Tesar, Pawel Kawalec

**Affiliations:** ^1^ Doctoral School of Medical and Health Sciences, Jagiellonian University Medical College, Cracow, Poland; ^2^ Department of Nutrition and Drug Research, Faculty of Health Sciences, Jagiellonian University Medical College, Cracow, Poland; ^3^ Agency for Medicines and Medical Devices of Bosnia and Herzegovina, Sarajevo, Bosnia and Herzegovina; ^4^ Romanian Association of International Medicine Manufacturers (ARPIM), Bucharest, Romania; ^5^ Department of Pharmacology and Clinical Pharmacology, Medical Faculty of the University of Montenegro, University of Montenegro, Podgorica, Montenegro; ^6^ Agency for Medicinal Products and Medical Devices (HALMED), Zagreb, Croatia; ^7^ Faculty of Biotechnology and Drug Development, University of Rijeka, Rijeka, Croatia; ^8^ Pharmacy and Pharmacology Center, Faculty of Medicine, Vilnius University, Vilnius, Lithuania; ^9^ Medison Pharma, Petah-Tiqva, Israel; ^10^ Department of Reimbursement, National Health Insurance Fund Management, Budapest, Hungary; ^11^ Department of Organization and Economics of Pharmacy, Faculty of Pharmacy, Medical University of Sofia, Sofia, Bulgaria; ^12^ Institute of Family Medicine and Public Health, University of Tartu, Tartu, Estonia; ^13^ Hungarian Health Economics Association, Budapest, Hungary; ^14^ Yerevan State Medical University, Yerevan, Armenia; ^15^ Department of Pharmacology with Clinical Pharmacology, Horbachevsky Ternopil National Medical University, Ternopil, Ukraine; ^16^ EconHealth s.r.o. Prague, Czechia; ^17^ Department of Pharmacy, Faculty of Medicine, University of Nis, Nis, Serbia; ^18^ Department of Organization and Management of Pharmacy, Faculty of Pharmacy, Comenius University in Bratislava, Bratislava, Slovakia

**Keywords:** CLL, targeted therapies, reimbursement, healthcare policy, drug accessibility

## Abstract

**Background:**

Despite recent therapeutic advances in chronic lymphocytic leukemia (CLL), access to innovative treatments may still be uneven outside Western Europe. This study aimed to explore reimbursement policy and access to novel targeted CLL therapies across selected countries and to analyze factors associated with differences in treatment availability and reimbursement timelines.

**Methods:**

Reimbursement frameworks, timelines, and accessibility of six novel CLL therapies were assessed across 15 countries in Central and Eastern Europe, the Balkans, Armenia, and Israel. Data were collected via expert surveys in late 2024, based on publicly available national and regional sources. The survey covered reimbursement extent, timelines, policy restrictions, coverage pathways, and health technology assessment (HTA) evaluations. Comparative analyses examined regional differences in reimbursement and their potential drivers. Spearman’s rank correlation was used to explore associations between the number of reimbursed therapies, reimbursement delays, and demographic, macroeconomic, and epidemiological variables.

**Results:**

The number of reimbursed therapies ranged from zero (Ukraine, Armenia) to five (Czech Republic), with a regional mean of 2.7 (SD = 1.38) and overall mean time to reimbursement of 29.3 months (SD = 21.4). Ibrutinib, reimbursed in 13 countries, had the longest mean reimbursement delay (35.6 months), while venetoclax (11 countries, 26.5 months), acalabrutinib (9 countries, 16.4 months), and zanubrutinib (6 countries, 15.2 months) had shorter delays. Gross domestic product (GPD) *per capita* showed a moderate positive correlation with the number of reimbursed therapies (ρ = 0.673, p = 0.006). Borderline significant associations were noted for CLL incidence and mortality (p = 0.050). Reimbursement indications were often restricted, particularly for patients without deletion 17p or TP53 mutations who experienced late relapses. Data on HTA outcomes and the number of treated patients were limited in several countries, and common challenges included funding constraints, administrative barriers, and the lack of centralized rare disease policies.

**Conclusion:**

Significant disparities in access to targeted CLL therapies persist across the analyzed countries, with the number of reimbursed therapies positively correlated with GDP *per capita*.

## 1 Introduction

Chronic lymphocytic leukemia (CLL) is one of the most common types of leukemia in adults, characterized by the accumulation of dysfunctional B lymphocytes in the blood, bone marrow, and lymphoid tissues (Eichhorst et al., 2021). It is the most prevalent leukemia in developed countries, accounting for 25%–30% of all leukemia cases in the United States (Siegel et al., 2021). Globally, the incidence of CLL reached approximately 103,467 cases in 2019, with 44,613 deaths, reflecting a significant increase of 155% and 107%, respectively, since 1990 (Ou et al., 2022). Despite its high prevalence in developed regions, CLL is classified as a rare disease, with an age-standardized incidence rate of 3.00 per 100,000 in Central Europe and 2.16 per 100,000 in Eastern Europe (Ou et al., 2022). The incidence of CLL increases significantly with age, with a median age at diagnosis of 70 years and a slightly higher prevalence in men than women (SEER, 2024). Geographic and racial disparities in CLL incidence are well-documented, with the disease being more common in developed regions, particularly among individuals of Caucasian descent and Jews of Eastern European origin, while being less common in Asian countries, such as China and Japan (Mukkamalla et al., 2025). These disparities likely reflect a combination of genetic, environmental, and healthcare-related factors (Mukkamalla et al., 2025), highlighting the importance of region-specific healthcare strategies.

CLL remains an incurable disease, with patients often requiring multiple lines of treatment throughout its course (Eichhorst et al., 2021). Heavily pretreated patients have the greatest unmet therapeutic needs, highlighting the necessity for more effective treatment options in this population (Mato et al., 2022). Recent advances in understanding CLL pathophysiology, especially the molecular pathways driving disease progression, have facilitated the development of innovative therapies targeting key mechanisms. These include Bruton’s tyrosine kinase inhibitors (BTKis), phosphoinositide 3-kinase inhibitors, and B-cell lymphoma two antagonists (BCL2a), which have significantly reshaped the treatment paradigm for CLL patients (Mato et al., 2022). Over the past decade, six new agents have been approved in Europe for the treatment of relapsed/refractory (R/R) CLL: ibrutinib, idelalisib, venetoclax, acalabrutinib, zanubrutinib, and duvelisib. These therapies have revolutionized CLL treatment by offering novel mechanisms of action, greater efficacy, and a more favorable safety profile compared to traditional chemoimmunotherapy (Monica et al., 2024a; 2024b). In addition, these new therapeutic options have given clinicians and patients greater flexibility in disease management, allowing for more personalized treatment strategies.

While advancements in targeted therapies have significantly improved patient outcomes, disparities in drug availability remain a pressing issue, particularly in Central and Eastern European (CEE) and Balkan countries. Reimbursement policies, shaped by diverse economic conditions, healthcare system capacities, and national drug evaluation frameworks, play a pivotal role in determining patient access to these innovative treatments (Kawalec et al., 2017). Although countries in the CEE region share certain historical and economic commonalities (Kawalec et al., 2017), their approaches to drug reimbursement and pricing differ significantly (Malinowski et al., 2018). Key factors such as gross domestic product (GDP) *per capita*, healthcare budgets, and the prioritization of health interventions often contribute to substantial variability in the availability of life-extending therapies. Additionally, differences in pharmaceutical expenditure levels, health technology assessment (HTA) requirements, and the strategic priorities of pharmaceutical companies further exacerbate inconsistencies in drug access across the region. These challenges are compounded by variations in orphan drug policies, reflecting the diversity of national legislation and strategies aimed at supporting research and development of new therapies (Jakubowski et al., 2024). Concerns such as high therapy costs, reliance on single-arm trials, and small sample sizes in clinical studies can hinder timely access to innovative drugs, as these limitations may complicate the HTA process (Zamora et al., 2019). Consequently, while some countries may fully reimburse essential treatments for all eligible CLL patients, others may impose restrictions based on clinical or economic criteria, potentially delaying or limiting access to these therapies.

This study aimed to identify disparities in the reimbursement of targeted therapies for CLL patients across the expanded group of analyzed CEE and Balkan countries. Specifically, it focused on assessing differences in the number of available treatments, reimbursed regimens, reimbursement indications, and their evolution across the included countries. Data on the number of treated patients were also collected to illustrate variations in access and utilization. Additionally, the study sought to determine whether demographic, macroeconomic, and epidemiological factors influence the number of reimbursed drugs, particularly by examining potential correlations with population size, life expectancy, the proportion of elderly individuals, GDP *per capita*, healthcare spending, and CLL-specific epidemiological indicators. Another objective was to analyze reimbursement delays and to explore their association with the above variables. Finally, the study compared funding channels, access mechanisms, official drug prices, and reimbursement expenditures, and reviewed HTA requirements and outcomes for targeted therapies in R/R CLL, with the aim to provide a comprehensive overview of the factors shaping access to CLL treatments in the region. The study was designed to provide valuable insights for policymakers, clinicians, and stakeholders, highlighting both challenges and opportunities in promoting equitable access to care and supporting efforts to improve health outcomes for CLL patients across Europe.

## 2 Methods

### 2.1 Study design

This cross-sectional, multi-country study evaluated national reimbursement policies for six targeted therapies—ibrutinib, idelalisib, venetoclax, acalabrutinib, zanubrutinib, and duvelisib—approved for the treatment of R/R CLL over the past decade. A mixed-methods approach combining quantitative and qualitative analyses was employed. Data were collected for the following European countries: Bosnia and Herzegovina, Bulgaria, Croatia, the Czech Republic, Estonia, Hungary, Lithuania, Montenegro, Poland, Romania, Serbia, Slovakia, and Ukraine. For a broader comparative perspective, data collection also included Armenia and Israel. All the above countries are associated with the CEE chapter of the International Society for Pharmacoeconomics and Outcomes Research and share regional challenges in healthcare policies, economic constraints, and drug access, making their inclusion relevant. Data were collected in the second half of 2024, with findings current as of 1 January 2025.

### 2.2 Data collection

Data were gathered using a structured survey distributed to national reimbursement experts, including policymakers, HTA specialists, and healthcare professionals with expertise in oncology drug access.

The questionnaire, which included both open-ended and close-ended questions specifically developed for this study, was divided into four sections:

1. Reimbursement Access to CLL Therapies: the most comprehensive section focused on collecting information about the availability and accessibility of reimbursement for novel therapies for R/R CLL;
2. HTA Evaluation: assessed whether HTA evaluations were conducted for these therapies and examined publicly available cost-effectiveness data;
3. General Reimbursement Policy: explored specific reimbursement policies, including legal requirements and willingness-to-pay (WTP) thresholds;
4. General Reimbursement Situation: collected perspectives on the overall reimbursement environment for CLL.

The survey was completed by experts based on publicly available national and regional data sources, including reimbursement lists, drug databases, and official reports from national health agencies, ministries of health, and reimbursement authorities. A comprehensive list of data sources, along with the questionnaire used for data collection, is provided in the (Supplementary Material).

Additional country-level data—including macroeconomic indicators (e.g., GDP *per capita*, healthcare expenditure), demographic information, and CLL epidemiological data—were obtained from publicly accessible databases such as the World Bank Open Data (World Bank Group, 2021), the World Health Organization (WHO) (World Health Organization, 2021), and the Global Burden of Disease repository (Institute for Health Metrics and Evaluation (IHME), 2022) (Supplementary Table S1).

### 2.3 Data analysis

Data were analyzed descriptively and comparatively to identify reimbursement trends and disparities across the studied countries. A qualitative review of survey responses yielded country-specific profiles, outlining reimbursement policies and HTA outcomes for R/R CLL therapies.

Key metrics assessed included reimbursement coverage, time to reimbursement, barriers to access, the number of treated patients, reimbursement criteria, financial expenditures, HTA requirements, and other factors affecting drug availability and use. Additionally, the average (mean) number of reimbursed drugs per region and reimbursement delay—defined as the time between European Medicines Agency (EMA) approval and the official reimbursement date in each country—were analyzed using descriptive statistics (mean, standard deviation, median, and range). Descriptive statistics were calculated only for countries in which the respective drug had been reimbursed by the cut-off date (1 January 2025). In addition, an exploratory Kaplan-Meier time-to-event analysis was conducted to include countries without a reimbursement decision, offering an alternative perspective on reimbursement delays.

Comparative analyses were conducted to assess regional differences and their potential drivers, including economic and policy-related factors. In addition, Spearman’s rank correlation analyses were performed to examine the relationship between the number of reimbursed drugs and reimbursement delays for each therapy with various demographic factors (population size, percentage of individuals aged 65 years or older, and life expectancy), macroeconomic indicators (GDP *per capita*, healthcare expenditures as a percentage of GDP, and domestic general government health expenditure), and CLL epidemiological data (e.g., incidence, prevalence, and mortality). Spearman’s correlation was selected as a conservative approach, given the relatively small sample size and the method’s minimal assumptions regarding linearity and normality. Statistical calculations were performed using PS IMAGO PRO version 10.

### 2.4 Ethical considerations

The study was approved by the Research Ethics Committee at Jagiellonian University Medical College (opinion no. 118.6120.71.2023), ensuring compliance with ethical standards for data collection and analysis.

## 3 Results

### 3.1 Reimbursement access for R/R CLL therapies

#### 3.1.1 Reimbursed drugs

##### 3.1.1.1 Number of reimbursed drugs

**FIGURE 1:**
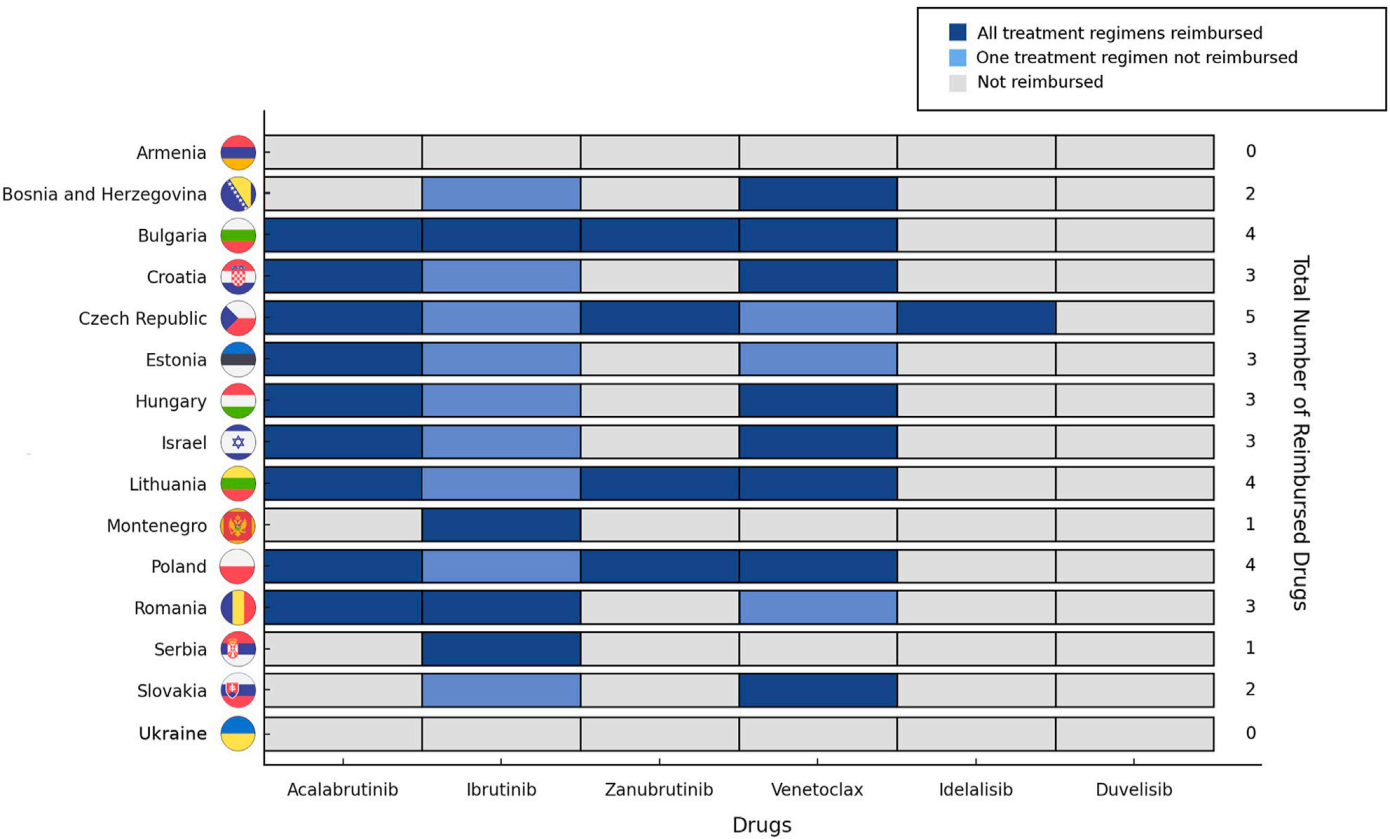
Reimbursement status of targeted therapies for R/R CLL across the analyzed countries.

The mean number of reimbursed therapies per country was 2.7 (SD = 1.38), reflecting a substantial variation in patient access to innovative treatments. The Czech Republic had the highest number of reimbursed therapies for R/R CLL (five of the six evaluated drugs), followed by Bulgaria, Lithuania, and Poland, each covering four therapies. The lowest availability was reported for Armenia and Ukraine (no reimbursed drugs), followed by Montenegro and Serbia (one drug), highlighting significant unmet needs for CLL patients in these regions. In the remaining countries, two to three therapies were reimbursed, typically including ibrutinib and venetoclax as a BCL2a with a different mechanism of action, with some countries extending reimbursement to acalabrutinib as an alternative BTKi (Figure 1).

The most widely reimbursed therapy was ibrutinib, available in 13 of the 15 countries. Venetoclax ranked second, with reimbursement in 11 countries, followed by acalabrutinib reimbursed in nine countries. The newest BTKi, zanubrutinib, was reimbursed in four countries, while idelalisib was covered in only one. Duvelisib was not reimbursed in any of the countries (Figure 1).

In the correlation analysis, the number of reimbursed drugs showed a moderate positive association with GDP *per capita* (ρ = 0.673, p = 0.006), suggesting that wealthier countries are more likely to finance a larger portfolio of targeted therapies for R/R CLL. Additionally, positive correlations were observed with CLL incidence and CLL-related deaths, although they were of borderline significance (both at p = 0.050). In contrast, other demographic, macroeconomic, and epidemiological variables did not show significant correlations with the total number of reimbursed drugs (Table 1).

**TABLE 1:** Spearman’s rank correlation between reimbursement metrics and selected demographic, macroeconomic, and epidemiological variables.

Variable	Number of reimbursed drugs		Reimbursement delayd					
			Acalabrutinib		Ibrutinib		Venetoclax	
	Spearman’s ρ	p-value	Spearman’s ρ	p-value	Spearman’s ρ	p-value	Spearman’s ρ	p-value
Demographic factors								
Population, number^a^	0.248	0.374	−0.283	0.460	−0.198	0.517	−0.319	0.339
Percentage of patients 65+, %^a^	0.380	0.163	0.017	0.966	0.357	0.231	0.232	0.492
Life expectancy, years^b^	0.338	0.218	−0.142	0.715	0.072	0.816	0.132	0.698
Macroeconomic indicators								
GDP *per capita*, US dollars^a^	0.673	0.006	0.067	0.865	−0.484	0.094	0.046	0.894
Current health expenditure, % GDP^b^	−0.449	0.093	−0.317	0.406	0.544	0.055	0.100	0.769
GGHE-D, % of GGE^b^	0.248	0.373	−0.100	0.797	0.476	0.100	0.434	0.183
CLL epidemiological data								
Incidence, ratio per 100,000^c^	0.513	0.050	0.333	0.381	−0.077	0.803	0.351	0.290
Prevalence, ratio per 100,000^c^	0.506	0.054	0.317	0.406	−0.006	0.845	0.337	0.311
Deaths, ratio per 100,000^c^	0.513	0.050	0.650	0.058	0.001	0.707	0.323	0.332
DALY, ratio per 100,00^c^	0.405	0.134	0.717	0.030	0.011	0.972	0.419	0.199

DALY, disability-adjusted life years; GDP, gross domestic product; GGE, general government expenditure; GGHE-D, domestic general government health expenditure; US, united states.

^a^ World Bank Open Data (2021).

^b^ World Health Organization (2021).

^c^ Institute for Health Metrics and Evaluation (2021) (Institute for Health Metrics and Evaluation (IHME), 2022).

^d^ A correlation analysis for the remaining therapies was not performed, as the number of data points available (i.e., countries with measurable reimbursement delays for those treatments) was insufficient for a meaningful statistical evaluation.

##### 3.1.1.2 Reimbursed regimens

Among the analyzed therapies, two drugs—ibrutinib and venetoclax—are approved for the treatment of R/R CLL, both as monotherapy and in combination with other agents. Ibrutinib can be used in a triple-drug regimen with bendamustine and rituximab, while venetoclax is approved for use in combination with rituximab. In most countries, ibrutinib was reimbursed only as monotherapy; however, Bulgaria, Montenegro, Romania, and Serbia also provided reimbursement for the combination regimen. Venetoclax as monotherapy and in combination with rituximab was reimbursed in eight countries (Bosnia and Herzegovina, Bulgaria, Croatia, Hungary, Israel, Lithuania, Poland, and Slovakia). In Romania, only venetoclax monotherapy was reimbursed, whereas in the Czech Republic, Estonia, and Hungary, reimbursement was limited to the venetoclax-rituximab regimen.

##### 3.1.1.3 Dates of reimbursement and reimbursement delays

The mean time to reimbursement for all therapies was approximately 26.4 months (SD = 17.1). However, significant variation in reimbursement timelines was observed across both therapeutic agents and countries (range: 1.5–91.3 months). In some cases, reimbursement was granted in less than 12 months, while in others, delays extended beyond 4 years (Table 2).

**TABLE 2:** Reimbursement delays for targeted therapies in R/R CLL across the analyzed countries.

Country/organisation	Date of issued decision (time from marketing authorization to reimbursement)						Reimbursement delay (country)	
	Acalabrutinib	Ibrutinib	Zanubrutinib	Duvelisib	Idelalisib	Venetoclax	Mean (SD)	Median [range], months
Marketing authorization in R/R CLL								
EMA	05-11-2020	21-10-2014	15-11-2022	19-05-2021	18-09-2014	05-12-2016	N/A	N/A
Initial reimbursement in R/R CLL								
Armenia	N/A	N/A	N/A	N/A	N/A	N/A	N/A	N/A
Bosnia and Herzegovina	N/A	07-05-2019 (54.5 months)	N/A	N/A	N/A	04-09-2020 (45.0 months)	49.8 (6.7)	49.8 [45.0–54.5]
Bulgaria	02-01-2022 (13.9 months)	02-01-2017 (26.4 months)	01-01-2024 (13.5 months)	N/A	N/A	01-01-2018 (12.9 months)	16.7 (6.5)	13.7 [12.9–26.4]
Croatia	15-04-2022 (17.3 months)	23-02-2017 (28.1 months)	N/A	N/A	N/A	01-03-2018 (14.8 months)	20.1 (6.9)	17.3 [14.8–28.1]
Czech Republic	01-11-2021 (11.9 months)	01-05-2019 (54.3 months)	01-11-2023 (11.5 months)	N/A	01-01-2018 (39.4 months)	01-03-2020 (38.8 months)	31.2 (18.9)	38.8 [11.5–54.5]
Estonia	01-07-2022 (19.8 months)	01-04-2017 (29.3 months)	N/A	N/A	N/A	01-07-2020 (42.8 months)	30.6 (11.6)	29.3 [19.8–42.8]
Hungary	16-10-2022 (23.3 months)	01-11-2016 (24.4 months)	N/A	N/A	N/A	15-01-2020 (37.3 months)	28.3 (7.8)	24.4 [23.3–37.3]
Israel	02-03-2021 (3.8 months)	15-01-2015 (2.8 months)	N/A	N/A	N/A	12-01-2017 (1.2 months)	2.6 (1.3)	2.8 [1.2–3.8]
Lithuania	19-05-2023 (30.4 months)	05-11-2016 (24.5 months)	19-09-2024 (22.1 months)	N/A	N/A	19-05-2019 (29.4 months)	26.6 (4.0)	27.0 [22.1–30.4]
Montenegro	N/A	29-12-2017 (38.3 months)	N/A	N/A	N/A	N/A	38.3 (0.0)	38.3 [38.3–38.3]
Poland	01-01-2023 (25.8 months)	01-09-2017 (34.3 months)	01-01-2024 (13.5 months)	N/A	N/A	01-01-2019 (24.8 months)	24.6 (8.5)	25.3 [13.5–34.3]
Romania	20-12-2020 (1.5 months)	27-12-2016 (26.2 months)	N/A	N/A	N/A	11-07-2018 (19.2 months)	15.6 (12.7)	19.2 [1.5–26.2]
Serbia	N/A	31-05-2022 (91.3 months)	N/A	N/A	N/A	N/A	91.3 (0.0)	91.3 [91.3–91.3]
Slovakia	N/A	01-03-2017 (28.3 months)	N/A	N/A	N/A	01-01-2019 (24.8 months)	26.6 (2.5)	26.6 [24.8–28.3]
Ukraine	N/A	N/A	N/A	N/A	N/A	N/A	N/A	N/A
Reimbursement delay (drug)								
Mean (SD), months	16.4 (9.7)	35.6 (21.3)	15.2 (4.7)	N/A	39.4 (0.0)	26.5 (13.8)	26.4 (17.1)	N/A
Median [range], months	17.3 [1.5–30.4]	28.3 [2.8–91.3]	13.5 [11.5–22.1]	N/A	39.4 [39.4–39.4]	24.8 [1.2–45.0]	N/A	24.8 [1.2–91.3]

EMA, european medicines agency; N/A, not applicable; SD, standard deviation.

Among the analyzed therapies, ibrutinib was the first drug to receive reimbursement, reflecting its earliest approval and adoption in certain markets. The fastest reimbursement was observed in Israel, where the process was completed in 2.8 months (15 January 2015) following EMA marketing authorization (21 October 2014). However, despite its early adoption in Israel, ibrutinib showed the longest reimbursement delays, with waiting times exceeding 24 months in all the remaining countries. The longest delays were recorded in Bosnia and Herzegovina (54.5 months), the Czech Republic (54.3 months), and Serbia (91.3 months). In contrast, second-generation BTKis, such as acalabrutinib and zanubrutinib, were reimbursed more quickly. Acalabrutinib demonstrated a mean reimbursement delay of 16.4 months (SD = 9.7), while zanubrutinib was reimbursed more quickly, with a mean delay of 15.2 months (SD = 4.7). Venetoclax exhibited a moderate reimbursement delay, with a mean time to reimbursement of 26.5 months (SD = 13.8). The fastest reimbursement of venetoclax was observed in Israel (1.2 months) and Croatia (14.8 months). However, several countries experienced substantial delays exceeding 24 months. These included Estonia, the Czech Republic, and Bosnia and Herzegovina, with reimbursement delays of 42.8, 38.8, and 45.0 months, respectively. Idelalisib was reimbursed exclusively in the Czech Republic, with a 39.4-month delay after its regulatory approval.

Disparities in reimbursement timelines were particularly striking at the country level. Israel consistently demonstrated the fastest access, with an average delay of 2.6 months, making it the only country in the region where multiple drugs were reimbursed within 12 months. Three countries—Croatia, Romania, and Bulgaria—achieved a moderate mean reimbursement time of 12–24 months for all drugs. In contrast, other countries faced delays exceeding 24 months. Poland fell just below the 24-month threshold, while the Czech Republic, Montenegro, and Estonia recorded delays ranging from 30 to 41 months. At the extreme end, two countries experienced delays exceeding 4 years, namely, Bosnia and Herzegovina (49.8 months) and Serbia (91.3 months).

Among the analyzed therapies, only acalabrutinib showed a significant positive correlation between reimbursement delays and disability-adjusted life years (ρ = 0.717, p = 0.030). None of the examined factors displayed a significant relationship with reimbursement timelines for ibrutinib or venetoclax (Table 1).

Exploratory Kaplan–Meier time-to-event curves illustrating time to reimbursement for each therapy are presented in Supplementary Figure S1.

#### 3.1.2 Reimbursed indications and target populations

##### 3.1.2.1 Limitations in reimbursed indications

An analysis of reimbursement restrictions across the analyzed countries revealed notable disparities in access to targeted therapies for R/R CLL. While a few countries, such as Bulgaria, Montenegro, and Romania, provide reimbursement consistent with registered indications, most other countries impose restrictions based on clinical characteristics, primarily patient mutational status and time to relapse (Supplementary Table S1).

In several countries, patients experiencing late relapse after prior therapy face restricted access to BTKis and venetoclax. For instance, Croatia and the Czech Republic exclude patients without deletion 17p (del17p) or TP53 mutation (mTP53) who relapse beyond 24 months. An exception is the Czech Republic, where patients with late relapses ineligible for further chemoimmunotherapy may still access these therapies. Similarly, Estonia, Lithuania, and Serbia restrict BTKi reimbursement for all patients relapsing beyond 36 months, regardless of their mutational status. However, Serbia allows access to ibrutinib for patients with late relapses who have undergone at least two prior lines of therapy. The strictest time-based restriction was observed in Slovakia, where reimbursement of a BTKi and venetoclax in combination with rituximab is unavailable for patients relapsing beyond 18 months. On the other hand, the most stringent population restrictions were observed in Hungary, where BTKis are reimbursed only for a highly selective group of patients. Eligibility is limited to those without del17p/mTP53 mutation who relapse after just one cycle of treatment, patients who have experienced at least two relapses or progressions within 12 months of their last relapse, and those with del17p/mTP53 or unmutated immunoglobulin heavy chain variable region who are ineligible for allogeneic stem cell transplantation. In Poland, venetoclax monotherapy is not reimbursed for patients without del17p or mTP53 or for those with these genetic abnormalities who were previously treated with a B-cell receptor (BCR) pathway inhibitor other than a BTKi (e.g., idelalisib).

Beyond time-based and mutational restrictions, some countries impose additional clinical eligibility criteria, often aligning with the populations examined in clinical trials, national guidelines for treatment initiation, or prior treatment history. In the Czech Republic, reimbursement for BTKis and venetoclax is limited to patients with an Eastern Cooperative Oncology Group performance status (ECOG PS) of 0–1. Similarly, Slovakia imposes additional hematologic criteria, requiring an absolute neutrophil count of at least 0.75 × 10^9^/L and a platelet count of at least 30 × 10^9^/L for BTKis and venetoclax + rituximab. In Poland, access to targeted therapies is regulated by strict inclusion criteria within the drug program, requiring patients to have an ECOG PS of 0–2 and to meet International Workshop on Chronic Lymphocytic Leukemia criteria for treatment initiation. Additional requirements include the absence of contraindications as specified in the Summary of Product Characteristics (SmPC), no active severe infections or significant comorbidities, and adequate organ function to ensure safe treatment initiation. Moreover, patients must confirm adherence to contraception guidelines where applicable.

Bosnia and Herzegovina limits ibrutinib reimbursement to patients with an ECOG PS of 0–1. However, patients with associated comorbidities are eligible if they have an ECOG PS of 0–2 and exhibit favorable prognostic characteristics, such as low tumor burden or a time to first metastasis of at least 18 months. In Croatia, BTKis and venetoclax are accessible only to patients meeting stringent diagnostic and disease severity criteria. Before initiating therapy, cytogenetic and fluorescence *in situ* hybridization testing, along with radiological assessment of lymph node, liver, and spleen involvement, are mandatory. Eligible patients must present with high-risk disease (Rai stage III–IV), a tumor mass ≥15 cm, progressive or symptomatic lymphadenopathy, or severe B symptoms that impact quality of life (e.g., unexplained weight loss ≥10% in the past 6 months, prolonged fever, or persistent night sweats). These criteria are consistent with established treatment guidelines for initiating therapy in CLL.

In Israel, while reimbursement covers all R/R CLL patients, eligibility depends on prior treatment history. To qualify for a BTKi or venetoclax, patients must have previously received one of the following regimens: bendamustine + rituximab, fludarabine + cyclophosphamide + rituximab, obinutuzumab, or chlorambucil with an anti-CD20 antibody. Furthermore, Israeli reimbursement policies impose specific sequential treatment limitations: patients are entitled to receive only one BTKi during their disease course, except for those treated with ibrutinib + venetoclax as a time-limited first-line regimen—these individuals may qualify for an additional BTKi in subsequent lines of therapy. Venetoclax is restricted to patients who have not previously been treated with this drug for their disease.

In addition to clinical restrictions, some countries imposed administrative limitations on reimbursed therapies. In Bulgaria, venetoclax is subject to a therapy monitoring program, restricting its administration to selected hospitals where treatment effectiveness is evaluated based on predefined criteria. This measure applies to innovative medicines that exceed cost-effectiveness thresholds or lack real-world evidence. Similarly, in the Czech Republic, all targeted therapies for R/R CLL are reimbursed exclusively in designated centers of excellence. In Slovakia, prescription restrictions are in place, allowing only specific healthcare providers to authorize reimbursed treatments.

##### 3.1.2.2 Changes to the scope of indications

In most analyzed countries, reimbursement indications for targeted therapies in R/R CLL have remained unchanged since their initial approval. However, notable exceptions include Israel and Poland, where criteria have evolved to expand access. In Israel, ibrutinib and venetoclax were initially reimbursed only for R/R CLL patients with del17p. However, these restrictions were lifted in 2016 and 2018, respectively, allowing their use irrespective of mutational status.

In Poland, the reimbursement policies for both ibrutinib and venetoclax have been gradually expanded. Initially, ibrutinib was reimbursed exclusively for R/R CLL patients with del17p/mTP53. In May 2019, reimbursement was extended to patients without del17p/mTP53 who met one of the following criteria: 1) relapse or treatment failure with venetoclax + an anti-CD20 antibody; 2) contraindications to this combination and either refractory disease or early relapse (≤24 months); or 3) treatment discontinuation due to toxicity. Venetoclax was initially reimbursed only as monotherapy for patients with del17p/mTP53 who had failed prior ibrutinib therapy. In November 2019, reimbursement was extended to cover venetoclax + rituximab for patients with del17p/mTP53, regardless of previous treatment, and for patients without del17p/mTP53 who were refractory to at least one line of chemoimmunotherapy or had early relapse (≤24 months). In November 2021, the reimbursement of combination therapy was broadened to cover all R/R CLL patients, irrespective of del17p/mTP53 status or time since last treatment. In January 2023, the reimbursement criteria for both ibrutinib and venetoclax were further expanded to their current scope.

##### 3.1.2.3 Number of treated patients

Data on the actual number of treated patients are largely limited across the region, with significant gaps in national reporting systems. In Bulgaria, Romania, Slovakia, and Montenegro, such data are not systematically collected, making it difficult to assess the true accessibility of targeted therapies.

In three countries (Czech Republic, Israel, and Serbia), patient numbers for at least one drug were available only as estimates derived from budget impact analyses or reimbursement submissions rather than from publicly available real-world data shared by healthcare systems (Table 3). These estimates provided insights into potential patient uptake but did not necessarily reflect actual treatment numbers. For example, Israel projected that approximately 132 new patients would receive ibrutinib annually, 40–46 would be started on acalabrutinib, and 38 would be initiated on venetoclax therapy. In the Czech Republic, budget impact analyses suggested that around 130 new patients would receive ibrutinib annually, 27 would begin venetoclax treatment, and approximately 57 would receive acalabrutinib. Additionally, zanubrutinib was projected to be initiated in 37 patients in 2024, increase to 51 in the second year and stabilize at 59 patients annually from the third year onward. Idelalisib was estimated to be prescribed to approximately 129 patients. Serbia provided an estimated range of 90–110 patients treated with ibrutinib, but no confirmed real-world data are available to verify this. However, based on total reimbursement costs and the annual cost of therapy, the expected number of patients across all ibrutinib indications is 61.

**TABLE 3:** Number of patients treated with targeted therapies across the analyzed countries.

Country	Reimbursed drug	Year							
		2017	2018	2019	2020	2021	2022	2023	2024
Bosnia and Herzegovina	Ibrutinib	NYR	NYR	ND	ND	ND	4	6	ND
	Venetoclax	NYR	NYR	NYR	10	13	21	25	35
Croatia	Acalabrutinib	NYR	NYR	NYR	ND	ND	18	ND	ND
	Ibrutinib	53	61	70	80	92	106	ND	ND
	Venetoclax	4	10	20	32	49	82	ND	ND
Czech Republic	Acalabrutinib	NYR	NYR	NYR	NYR	ND	∼57 new patients annually^a^		
	Ibrutinib	NYR	NYR	∼59^a^	∼101^a^	∼124^a^	∼128^a^	∼130^a^	ND
	Zanubrutinib	NYR	NYR	NYR	NYR	NYR	NYR	ND	∼37^a^
	Venetoclax	NYR	NYR	NYR	∼27 new patients annually^a^				
	Idelalisib	NYR	NYR	∼59^a^	∼101^a^	∼124^a^	∼128^a^	∼129^a^	ND
Estonia	Acalabrutinib	NYR	NYR	NYR	NYR	NYR	15	41	62
	Ibrutinib	ND	ND	ND	ND	82	77	67	96
	Venetoclax	NYR	NYR	NYR	ND	40	73	82	105
Hungary	Acalabrutinib	NYR	NYR	NYR	NYR	NYR	ND	<10	27
	Ibrutinib	218	321	414	490	571	553	508	425
	Venetoclax	NYR	NYR	NYR	87	145	233	379	488
Israel	Acalabrutinib	NYR	NYR	NYR	NYR	∼40–46 new patients annually^a^			
	Ibrutinib	∼132 new patients annually^a^							
	Venetoclax	∼38 new patients annually^a^							
Lithuania	Acalabrutinib	NYR	NYR	NYR	NYR	NYR	NYR	98	ND
	Ibrutinib	NYR	NYR	NYR	NYR	ND	ND	362	ND
	Zanubrutinib	NYR	NYR	NYR	NYR	NYR	NYR	NYR	7
	Venetoclax	NYR	NYR	11	37	154	94	111	218
Poland	Acalabrutinib	NYR	NYR	NYR	NYR	NYR	NYR	582	1,029
	Ibrutinib	0	252	494	572	711	767	1,116	1,267
	Zanubrutinib	NYR	NYR	NYR	NYR	NYR	NYR	NYR	594
	Venetoclax	NYR	NYR	70	320	644	1,388	1,749	1,830
Serbia	Ibrutinib	NYR	NYR	NYR	NYR	NYR	∼90–110^a^	ND	ND

ND, no data; NYR, not yet reimbursed.

^a^ Estimated number of patients included in the budget impact analysis or reimbursement submission.

The real-world patient numbers include both monotherapy and combination therapy, and may also encompass first-line patients.

Real-world patient data were available in six countries (Bosnia and Herzegovina, Croatia, Estonia, Hungary, Lithuania, and Poland). The total number of treated patients generally increased over time in these countries; however, reported data may also include patients receiving treatment in earlier lines of therapy or under alternative reimbursement indications. In Poland, the number of ibrutinib-treated patients increased from 252 in 2018 to 1,267 in 2024, while venetoclax use expanded from 70 patients in 2019 to 1,830 in 2024 (includes patients receiving these drugs as first-line treatment). A similar trend was observed in Hungary, where ibrutinib use grew from 218 patients in 2017 to a peak of 571 in 2021, before slightly declining to 425 in 2024. The number of venetoclax-treated patients steadily increased from 87 in 2020 to 488 in 2024, while acalabrutinib use was reported in fewer than 10 patients in 2023 but expanded to 27 in 2024.

In contrast, patient numbers in Bosnia and Herzegovina remained particularly low, suggesting that reimbursement was granted on a case-by-case basis rather than through a structured national program. For instance, only four patients were treated with ibrutinib in 2022, increasing to six in 2023. Venetoclax was reimbursed for 10 patients in 2020, with coverage gradually expanding to 25 by 2023 (including both monotherapy and combination with rituximab). In other countries, a gradual increase in patient numbers was also observed following reimbursement. In Lithuania, 98 patients received acalabrutinib in 2023, while in Estonia, the number of venetoclax-treated patients increased from 40 in 2021 to 82 in 2023. Croatia also reported a steady growth, with ibrutinib use increasing from 53 patients in 2017 to 106 in 2022.

#### 3.1.3 Reimbursement pathways and costs

##### 3.1.3.1 Funding channels and access mechanisms

In most countries, reimbursed therapies are covered under standard mechanisms within the National Health Insurance system, without the implementation of innovative or dedicated reimbursement schemes or instruments for rare diseases or oncology treatments. For example, reimbursement is provided either through the basic reimbursed drug list (e.g., Croatia, Serbia, Israel, Slovakia, Estonia, Bulgaria, Lithuania) or through standard drug programs included in the Ministry of Health’s reimbursed drug list (e.g., Poland: Drug Program for CLL – B.79, Romania: Oncology Program P3). In Hungary, an itemized reimbursement program is available for selected healthcare providers (specific hospitals).

A notable exception is Montenegro, where ibrutinib is reimbursed under the “Treatment of Rare Diseases and Implementation of Transplantation Programs” rather than through a general reimbursement scheme. Additionally, Bosnia and Herzegovina imposes administrative restrictions on the reimbursement of venetoclax. As monotherapy, venetoclax is covered under a specific list of medicines applied in limited quantities and is reimbursed only in one entity (the Republic of Srpska). For combination therapy, it is not directly reimbursed but rather secured through a national health insurance tender procedure based on hospital estimations. In Romania, ibrutinib and venetoclax are reimbursed via the orphan drug pathway.

In almost all analyzed countries, reimbursement decisions were granted indefinitely. The only exception was Poland, where the initial reimbursement decision is valid for 2 years, requiring the marketing authorization holder to apply for an extension thereafter.

##### 3.1.3.2 Official drug prices and reimbursement expenditures

All analyzed drugs are fully reimbursed across the studied countries (reimbursement level: 100%) with no out-of-pocket costs, except Estonia, where patients are required to pay an additional out-of-pocket cost of €3.50 (effective 1 January 2025; previously €2.50).

The reimbursement prices of targeted therapies for R/R CLL vary significantly between countries, reflecting differences in national pricing policies, reimbursement regulations, and market conditions. Additionally, some countries have implemented price adjustments since the initial reimbursement decision, leading to notable price reductions in certain markets (Supplementary Table S2).

The comparison of monthly therapy costs based on official list prices showed considerable variation for acalabrutinib. The highest monthly therapy cost was observed in Israel (6,440.86 EUR), Bulgaria (6,066.24 EUR), and Poland (6.181.03 EUR). In contrast, the lowest costs were recorded in Lithuania (3,148.83 EUR) and the Czech Republic (4,482.83 EUR) following a 12% price reduction since the initial reimbursement decision. Notably, no price increases for this therapy were reported across the analyzed countries.

For ibrutinib, the highest monthly therapy cost based on official list prices was reported in Israel (6,112.26 EUR), followed by Estonia (6,032.58 EUR). The most significant price reductions since the initial reimbursement decision were observed in Israel (−15%) and Montenegro (−12%). Price reductions were also implemented in Bulgaria (−10%), Bosnia and Herzegovina (−7%), Lithuania (−6%), Poland (−6%), and Romania (−5%).

In the case of zanubrutinib, the highest monthly therapy cost was observed in Poland (5,405.77 EUR), followed by Bulgaria (4,940.03 EUR), while Lithuania showed the lowest cost (3,074.52 EUR). No price changes were reported for zanubrutinib in any country except Lithuania, where a 3% price reduction was noted since the initial decision.

For venetoclax, the highest monthly therapy cost was reported in Estonia (6,407.84 EUR), while the lowest cost was found in Lithuania (3,944.70 EUR). The drug has undergone significant price reductions in several markets, including Bulgaria (−25%), Israel (−23%), Lithuania (−32%), Poland (−23%), and Romania (−22%), reflecting substantial price adjustments since the initial reimbursement decision.

Idelalisib, which is reimbursed exclusively in the Czech Republic, has a monthly therapy cost of 4,074.26 EUR.

Publicly available data on total pharmacotherapy reimbursement budgets and annual expenditures for specific therapies were limited in many countries. Among the analyzed countries, Poland (5.23 billion EUR) and the Czech Republic (estimated 3.98 billion EUR) had the highest overall pharmacotherapy budgets, while Montenegro (0.12 billion EUR) and Estonia (0.19 billion EUR) had the lowest. In most cases, annual reimbursement expenditures for individual therapies did not exceed 1% of the total pharmacotherapy reimbursement budget. Notable exceptions included ibrutinib in Croatia (21.85 million EUR, 1.69%), Lithuania (13.31 million EUR, 2.5%), and Estonia (2.17 million EUR, 1.14%) (Table 4).

**TABLE 4:** Total pharmacotherapy reimbursement budgets and annual reimbursement expenditures across the analyzed countries.

Country	Year	Estimated yearly pharmacotherapy expenditures	Total yearly reimbursement expenditures (% of total expenditures)		
			Acalabrutinib	Ibrutinib	Venetoclax
Armenia	N/A	Not publicly available	Not reimbursed	Not reimbursed	Not reimbursed
Bosnia and Herzegovina	2023	€0.50B	Not reimbursed	€0.27M (<0.01%)	€0.56M (<0.01%)
Bulgaria	N/A	Not publicly available	Not publicly available	Not publicly available	Not publicly available
Croatia	2022	€1.29B	€1.12M (0.09%)	€21.85M (1.69%)	€6.05M (0.47%)
Czech Republic	2022	€3.98B (est.)	Not publicly available	Not publicly available	Not publicly available
Estonia	2022	€0.19B	€0.40M (0.21%)	€2.17M (1.14%)	€1.69M (0.89%)
Hungary	2024	€1.90B	Not publicly available	Not publicly available	Not publicly available
Israel	N/A	Not publicly available	Not publicly available	Not publicly available	Not publicly available
Lithuania	2023	€0.53B	€1.50M (0.29%)	€13.31M (2.5%)	€4.55M (0.81%)
Montenegro	2022	€0.12B	Not reimbursed	Not publicly available	Not reimbursed
Poland	2023	€5.23B	€7.70M (0.15%)	€19.31M (0.37%)	€38.82M (0.74%)
Romania	2024	€3.54B	Not publicly available	Not publicly available	Not publicly available
Serbia	2022	€1.49B	Not reimbursed	€3.79M (0.26%)	Not reimbursed
Slovakia	2023	€1.51B	Not reimbursed	Not publicly available	Not publicly available
Ukraine	N/A	Not publicly available	Not reimbursed	Not reimbursed	Not reimbursed

Data for other drugs were excluded due to a lack of publicly available information.

B = billion; M = million N/A = not available.

### 3.2 HTA for R/R CLL drugs

Publicly available data on HTA evaluations and economic assessments for targeted therapies in R/R CLL were limited in many countries. In Israel, Armenia, Croatia, and Bosnia and Herzegovina, no HTA evaluations were conducted for any of the analyzed drugs. In Montenegro, HTA is required, but the results are not publicly available. In Hungary, HTA evaluations were performed for acalabrutinib, venetoclax + rituximab, venetoclax monotherapy, zanubrutinib, and ibrutinib, but the results for the last four are unavailable. In Serbia, a complex HTA is not required; however, a financial assessment was conducted for ibrutinib, although the results are not publicly available. Romania follows a simplified HTA process, requiring a cost comparison between the new medicine and the least expensive reimbursed alternative for the same indication, with orphan drugs exempt from this requirement. If no therapeutic alternatives exist, cost comparison is also not required.

In Bulgaria, the Czech Republic, Estonia, Poland, and Slovakia, HTA data are at least partially publicly available. Similarly, in Ukraine, HTA conclusions and recommendations are publicly available, except confidential information or restricted parts of HTA dossiers (Supplementary Table S3).

In Bulgaria, acalabrutinib and venetoclax (both as monotherapy and in combination with rituximab) were found to be cost-effective, while ibrutinib and zanubrutinib were not. However, incremental cost-utility ratio (ICUR) results were not disclosed in the publicly available reports.

In the Czech Republic, a cost-minimization analysis was performed for acalabrutinib. Ibrutinib was found not to be cost-effective compared to temsirolimus and best supportive care, with ICUR values of 71,656 EUR/QALY and 218,949 EUR/QALY, respectively. On the other hand, idelalisib + rituximab was found to be cost-effective compared to rituximab alone, with an ICUR of 31,146 EUR/QALY. Venetoclax + rituximab was also demonstrated to be cost-effective relative to ibrutinib, with an ICUR of 13,363 EUR/QALY when compared to idelalisib + rituximab.

In Estonia, an HTA analysis for acalabrutinib was conducted using a cost-minimization approach relative to ibrutinib and venetoclax. For ibrutinib, a QALY gain was observed compared to chlorambucil, as was the case for venetoclax compared to ibrutinib. However, no ICUR values were reported.

In Poland, cost-effectiveness ratios were generally not disclosed. Exceptions included ibrutinib *versus* ofatumumab, which was found not to be cost-effective (67,792 EUR/QALY), and ibrutinib *versus* bendamustine + rituximab (€63,280 EUR/QALY). Venetoclax monotherapy was also not cost-effective compared to best supportive care, with ICUR values of 48,256 EUR/QALY for the post-ibrutinib population and 50,267 EUR/QALY for the post-BCRi population. Similarly, idelalisib + rituximab was not cost-effective, with ICUR values ranging from 147,271 EUR/QALY to 166,061 EUR/QALY, depending on the comparator.

In Ukraine, HTAs were conducted for ibrutinib and venetoclax + rituximab. The results for ibrutinib are not publicly available. Venetoclax + rituximab was not found to be cost-effective, with an ICUR value of 55,579.37 EUR/QALY compared to bendamustine + rituximab.

No data are available on whether additional analyses incorporating risk-sharing schemes (RSS) were conducted in Bulgaria. In the Czech Republic, Poland, and Slovakia, RSS-based versions of HTA reports were usually prepared; however, both the specific details of the RSS and the resulting cost-effectiveness outcomes remain confidential. In the Czech Republic, it is likely that reimbursement was limited by confidential discounts and budget caps. In Estonia, no RSS-based versions of the assessments were implemented. In Ukraine, the HTA conclusion for venetoclax + rituximab included a recommendation for a managed entry agreement, based on identified uncertainties and suggested ranges of cost-effective prices.

The time from HTA results to reimbursement was under 6 months in Bulgaria and Slovakia, while in Estonia and Hungary, the process typically took 6 months to 1 year (with some exceptions). In Poland, reimbursement for acalabrutinib, ibrutinib, and venetoclax monotherapy took one to 2 years, whereas zanubrutinib was reimbursed in less than 6 months.

### 3.3 General reimbursement policy and systemic perspectives

#### 3.3.1 General reimbursement policy in analyzed countries

##### 3.3.1.1 Reimbursement policies for hemato-oncological and orphan drugs

In most of the analyzed countries, hemato-oncological therapies are reimbursed through the standard national reimbursement policy based on a national reimbursement list. However, there are exceptions to this approach. Bosnia and Herzegovina operates a distinct reimbursement system, where drugs are included in separate lists, such as the Entity of Republic of Srpska’s reimbursement list and the Solidarity Fund list. Medications from these lists are typically procured through tenders, with an annual supply system in place. Ukraine, on the other hand, employs a centralized procurement program for oncological and hematological therapies. Rather than integrating these treatments into a standard reimbursement list, the country follows a dedicated program that centralizes purchasing and distribution. In Bulgaria, hemato-oncological therapies are financed through a separate budget within the National Health Insurance Fund.

The reimbursement approach for orphan drugs varies across countries, with some implementing specific policies while others lacking dedicated frameworks. In Armenia, Bulgaria, Croatia, Estonia, Israel, and Ukraine, there are no special reimbursement considerations for orphan drugs, and they are subject to the same evaluation criteria as other pharmaceuticals. In Armenia, CLL is not even recognized as a rare disease and is not covered by any specific reimbursement policies. In Bosnia and Herzegovina, reimbursement for rare and orphan drugs is possible through specific reimbursement lists available in local healthcare funds (either at the entity or cantonal level). There are no strict predefined requirements, and access to orphan drugs is typically subject to negotiations with the government. Some countries, such as Hungary and Slovakia, provide higher WTP thresholds for orphan drugs, while in the Czech Republic, the WTP threshold may be exceeded.

Other countries, like Montenegro and Serbia, allow for individual reimbursement of orphan drugs. In Montenegro, the special commission of Ministry of Health can approve the reimbursement of an orphan drug for an individual patient, even if it is not officially reimbursed, based on a proposal from the council of specialists in certain disciplines. In Serbia, while orphan drugs are not automatically covered, a special request must be submitted to the Republic Health Insurance Fund’s Commission for Rare Diseases by a specialist treating the patient.

Romania and Poland use a more structured approach. Until 2020, Romania employed a fast-track, simplified assessment based solely on orphan drug status and the number of European Union (EU) member states where the medicine was reimbursed. Additionally, orphan drugs that have undergone a clinical trial in Romania or have been provided through a compassionate use program covering at least 50% of the eligible population for 1 year may qualify for unconditional reimbursement. In Poland, oncological and orphan drugs may be reimbursed through an accelerated pathway for highly innovative therapies. However, this requires that the drug has been registered for the given indication in the preceding year and is included in the official list published by the Polish HTA agency and the Ministry of Health. Under this pathway, submitting a full HTA report is not required—only a budget impact analysis needs to be provided.

Access to innovative therapies is generally determined on a case-by-case basis in most countries, including Armenia, Bosnia and Herzegovina, Bulgaria, Croatia, Hungary, Israel, Slovakia, and Ukraine. However, some countries have established specific programs to support innovative or high-value therapies. In Hungary, there is a possibility for reimbursement through a Named Patient Program for drugs with strong clinical evidence. Romania has structured pathways for the evaluation and reimbursement of highly innovative therapies, assessed by national HTA bodies. Targeted oncological therapies are reimbursed within the oncology program under the C2 reimbursement list of the National Health Insurance House. To qualify, a therapy must achieve a minimum score of 60–79 out of 100 points, which allows for conditional reimbursement, subject to a risk-sharing financial agreement. A score of 80 or more enables unconditional reimbursement, which means that the medicine does not require a risk-sharing agreement but must have a defined therapeutic and prescription protocol and be published in national legislation. In Montenegro and Serbia, specific provisions and programs exist to facilitate access to innovative or targeted therapies. In Ukraine, access to innovative therapies is also facilitated through the use of managed entry agreements. In the Czech Republic and Estonia, there are no specific reimbursement considerations for innovative targeted therapies in oncology and hematology.

Of the additional orphan drug pathways available in various countries, only Romania has applied a specific approach to the reimbursement of orphan therapies for R/R CLL.

Beyond standard reimbursement mechanisms, additional support programs exist in several countries to improve access to therapies. In Estonia, private-public funds for cancer patients provide financial assistance beyond standard reimbursement. Poland also allows for individual reimbursement as an emergency access option for patients who have exhausted all available therapeutic options. In Romania, the “last resort treatment” program allows pharmaceutical companies to fund treatment for a limited number of patients before the EMA grants final approval or marketing authorization. Additionally, individual pharmaceutical companies may run support programs for specific pathologies. Slovakia has an extraordinary reimbursement regime (Löblová et al., 2019) that enables statutory health insurance funds to approve reimbursement for individual patients receiving drugs not included in the national positive drug list, similar to the individual funding request system in the United Kingdom (UK).

##### 3.3.1.2 Reimbursement and HTA requirements

**FIGURE 2:**
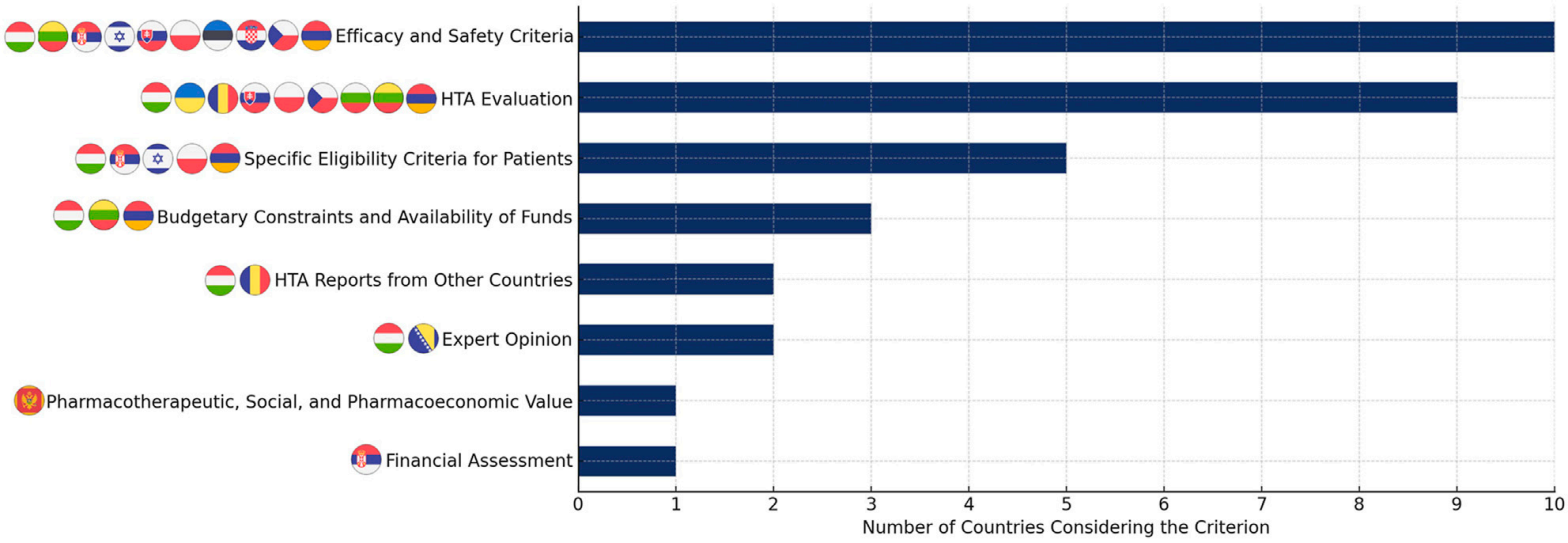
Criteria considered in the drug reimbursement process across the analyzed countries.

According to the survey, efficacy and safety (10 countries) and HTA evaluation (9 countries) are the most frequently considered criteria in the reimbursement process across most countries (Figure 2). Additionally, five countries (Armenia, Hungary, Israel, Poland, and Slovakia) apply specific eligibility criteria for patients to determine reimbursement eligibility. Beyond these factors, some countries incorporate additional considerations: Armenia, Lithuania, and Hungary take into account budgetary constraints and the availability of funds, while Bosnia and Herzegovina requires a pharmacological opinion from an expert in the field as a basis for evaluation, particularly within the Entity of Srpska. In Montenegro, pharmacotherapeutic value, social (public health) value, and pharmacoeconomic value play a role in reimbursement decisions. Romania integrates HTA reports from France, the UK (National Institute for Health and Care Excellence, Scottish Medicines Consortium), and Germany, along with the reimbursement status in EU member states and the UK. A minimum of three countries is required for a medicine to receive points, while those reimbursed in more than 14 countries receive the highest score. Serbia includes financial assessments as part of the reimbursement process.

The HTA process is required for reimbursement in most analyzed countries, including Bulgaria, the Czech Republic, Estonia, Hungary, Lithuania, Montenegro, Poland, Romania, Slovakia, and Ukraine. However, in Montenegro, while HTA is formally mandated, the details of the process are not publicly available. In Serbia, a simplified HTA approach is applied, primarily focusing on financial analysis, incorporating elements of cost-effectiveness and budget impact assessment. In Romania, HTA is required for most medicines, with the exception of contraceptives and vaccines, which are exempt from the evaluation process.

The duration of the HTA process varies significantly across countries, ranging from 50 days in Hungary to 10–12 months in the Czech Republic. Among the countries that require HTA, Estonia, Romania, and Serbia do not have a defined WTP threshold. In Poland and Bulgaria, the WTP threshold is set at 3× GDP *per capita* per QALY, while in the Czech Republic, a fixed value of 1.2 million CZK per QALY is applied. In Hungary, Slovakia, Lithuania, and Ukraine, the WTP threshold depends on the clinical benefit (QALY gain) of the assessed drug or the burden of disease. Additionally, in Slovakia and Hungary, alternative multipliers are used for orphan drugs, resulting in higher WTP thresholds (Table 5).

**TABLE 5:** HTA requirements in the analyzed countries.

Country	HTA required	Duration of HTA process	Willingness-to-pay
Armenia	No	N/A	N/A
Bosnia and Herzegovina	No	N/A	N/A
Bulgaria	Yes	180 days	3×GDP *per capita*/QALY
Croatia	No	N/A	N/A
Czech Republic	Yes	300–365 days	1,200,000.00 CZK/QALY (∼47,770 EUR/QALY)
Estonia	Yes	180–365 days	Not defined
Hungary	Yes	50 days	Variable^b^
Israel	No	N/A	N/A
Lithuania	Yes	180 days^a^	Variable^c^
Montenegro	Yes	Not publicly available	Not publicly available
Poland	Yes	180 days^a^	3×GDP *per capita*/QALY
Romania	Yes	90 days^a^	Not defined
Serbia	No (only financial assessment)	90–120 days (for financial assessment)	Not defined
Slovakia	Yes	130 days	Variable^d^
Ukraine	Yes	110 days	Variable^e^

^a^ Legal timeframe, real times are often longer (in Romania: possible stop-clocks and delays; currently, the real-time process takes 4–5 months, depending on complexity and the requested documentation).

^b^ The cost-effectiveness threshold varies between orphan and non-orphan drugs. For non-orphan drugs, the threshold is determined using a baseline GDP, *per capita* multiplied by a fixed factor, based on the surplus additional health gain, calculated as (QALY, of the tested technology–QALY, of the comparator)/QALY, of the tested technology. If the surplus additional health gain falls between 0.00 and 0.25, the threshold is 1.5× GDP, *per capita*; for values between 0.25 and 0.60, it increases to 2× GDP, *per capita*; and for gains between 0.60 and 1.00, the threshold reaches 3× GDP, *per capita*. For orphan drugs, an additional GDP, multiplier is applied to threshold, depending on the QALY, gain: for ∆QALY, 0.5, the multiplier is 3×; for ∆QALY, 1.0, it is 3.2×; for ∆QALY, 5.0, it increases to 4.6×; for ∆QALY, 10.0, it reaches 6.4×; for ∆QALY, 15.0, it is 8.2×; and for ∆QALY, 20.0, the highest multiplier of 10× is applied.

^c^ In Lithuania, the willingness-to-pay threshold varies with the burden of illness: 1× GDP, *per capita* (burden of disease: 0.1–0.49), 3× GDP, *per capita* (burden of disease: 0.5–0.74), and 5× GDP, *per capita* (burden of disease: 0.75–1).

^d^ The cost-effectiveness thresholds in Slovakia are based on QALY, gain compared to an alternative medical intervention. For standard treatments, the threshold is 2× GDP, *per capita* if the QALY, gain is greater than 0 and less than 0.33, and 3× GDP, *per capita* if the QALY, gain is 0.33 or more. For orphan drugs or innovative treatments, the thresholds are set at 3× GDP, *per capita* for a QALY, gain between 0 and 0.33, 5× GDP, *per capita* for a QALY, gain between 0.33 and 0.5, and 10× GDP, *per capita* for a QALY, gain of 0.5 or more.

^e^ Cost-effectiveness spending thresholds: Very efficient spending is defined as costs below 1× GDP, *per capita*, efficient spending falls within 1–3× GDP, *per capita*, low-efficiency spending is categorized as 3–5× GDP, *per capita*, and inefficient spending refers to costs exceeding 5× GDP, *per capita*.

##### 3.3.1.3 Frequency of reimbursement list updates

**FIGURE 3:**
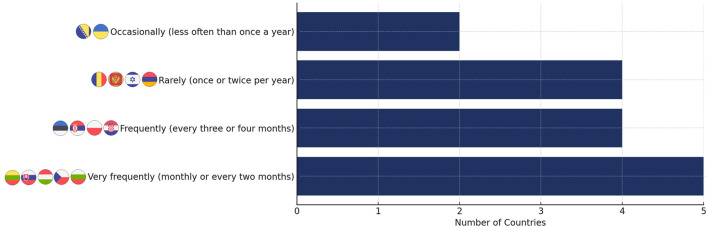
Reimbursement list update frequency across the analyzed countries.

The frequency of updates to reimbursement lists varies significantly across countries. In Bulgaria, the Czech Republic, Hungary, Lithuania, and Slovakia, the lists are updated very frequently, every month or two. In contrast, Armenia, Israel, Montenegro, and Romania revise their lists rarely, typically once or twice per year. Croatia, Estonia, Poland, and Serbia fall in the middle, updating their lists approximately every three to 4 months. Bosnia and Herzegovina and Ukraine update their reimbursement lists least frequently, less than once a year (Figure 3).

#### 3.3.2 Challenges, barriers, and potential solutions

Representatives from three surveyed countries, namely, Bosnia and Herzegovina, Romania, and Serbia, described their current reimbursement system as unfair for patients with CLL. In Bosnia and Herzegovina, inequities arise due to a lack of clearly defined requirements for listing medicines across the country. Reimbursement policies vary between the two entities, leading to unequal access to medicines. In Romania, the system was deemed unfair as HTA evaluations do not consider unmet medical needs or the degree of innovation, leading to extended waiting times for some medicines to be reimbursed. Similarly, in Serbia, the system was criticized for failing to reimburse most CLL therapies.

Several challenges and barriers to access were also identified. In Bosnia and Herzegovina, access to therapy differs between the two entities: venetoclax monotherapy is available in the Federation of Bosnia and Herzegovina, whereas in the Republic of Srpska, only the combination therapy is reimbursed. In Poland, a separate HTA submission is required for each combination therapy, even if its individual components are already reimbursed for the same indication (e.g., bendamustine + rituximab and ibrutinib are reimbursed, but the triple combination of ibrutinib + bendamustine + rituximab would require a new HTA and reimbursement submission). In Romania, insufficient laboratory capacity for molecular testing was identified as a key challenge, leading to delays in obtaining test results. Additionally, for medicines reimbursed through hospitals, delays in securing financing can result in suboptimal treatment schemes or longer waiting times for patients before starting the next line of therapy. In Ukraine, the ongoing war and limitations in healthcare funding were highlighted as major barriers to access.

**FIGURE 4:**
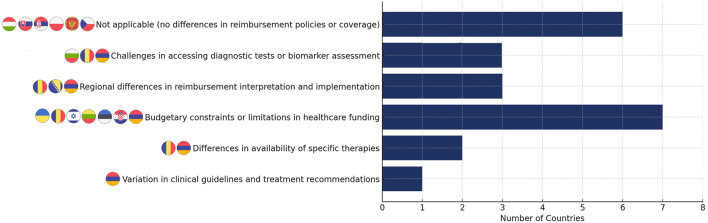
Factors potentially contributing to unequal access to CLL therapies.

Regarding factors potentially contributing to unequal access to CLL therapy for different subtypes or stages, the most frequently identified issue was budgetary constraints or limitations in healthcare funding, reported by Armenia, Croatia, Estonia, Israel, Lithuania, and Ukraine (Figure 4). Another common challenge was the variation in how reimbursement criteria were interpreted and implemented by different regions or authorities, as highlighted by Bosnia and Herzegovina, Armenia, and Romania. Additionally, difficulties in accessing or obtaining diagnostic tests or biomarker assessments for precise CLL subtype or stage determination were reported in Bulgaria, Romania, and Armenia. Romania and Armenia also noted differences in the availability of specific therapies or targeted agents for different CLL subtypes or stages. In the past, variations in clinical guidelines and treatment recommendations were reported in Armenia, where inconsistencies in guidelines were identified as a barrier to equitable access. However, as of July 2024, Armenia adopted its first national clinical standard protocol for the management of CLL in adults, approved by the Ministry of Health, which may help address these previously reported inconsistencies. Additionally, both Armenia and Hungary highlighted disparities in clinical evidence supporting the effectiveness of treatments for specific subgroups or stages.

Regarding challenges in the reimbursement process, the Czech Republic reported that the reimbursement of venetoclax + rituximab took 2 years due to issues with the selected comparator and failure to meet the cost-effectiveness threshold. In Poland, most HTA evaluations for CLL therapies received negative HTA recommendations, significantly extending the reimbursement process. These negative recommendations were primarily due to the lack of direct comparative evidence. In Romania, the overall underfunding of the healthcare system has resulted in significantly longer reimbursement timelines for certain medicines compared to those previously reimbursed. In Slovakia, delays were attributed to pharmaceutical companies prioritizing larger and wealthier markets, leading to postponements in the availability of CLL drugs. In Ukraine, drug shortages may occur due to budget and procurement constraints, logistical challenges, and the ongoing war, further complicating access to essential therapies.

Several countries suggested potential improvements to the reimbursement system. In Armenia, proposals included expanding the reimbursement list to include innovative therapies, developing subsidy programs for diagnostic tests, and allocating additional funds for diseases such as CLL. In Bosnia and Herzegovina, stakeholders emphasized the need to harmonize the reimbursement process across the two entities to ensure consistent criteria and equal access to medicines. They also recommended implementing HTA at all levels of healthcare funds for specific therapies. Romanian representatives advocated for a clearer reimbursement process with defined timelines, particularly given the requirement to update the reimbursement list at least once per year if sufficient budget is available. Additional recommendations included implementing a horizon scanning process to anticipate budget needs for medicines expected to undergo HTA and introducing an early access program to enable patient-by-patient access from EMA approval until national reimbursement—similar to the Temporary Authorization for Use program (Autorisation Temporaire d’Utilisation) in France or the Innovation Fund in Italy and the UK. In Serbia, a key recommendation was to increase the number of CLL drugs included in the positive list.

## 4 Discussion

This study highlights the significant variability in reimbursement patterns, HTA requirements, and timelines for novel CLL therapies across analyzed countries. While targeted therapies such as BCRi and BCL2a have revolutionized CLL management over the past decade, access to these treatments continues to differ widely between countries.

A key observation is the considerable heterogeneity in the number of reimbursed R/R CLL therapies among the analyzed countries. While the Czech Republic, Bulgaria, Lithuania, and Poland reimburse multiple targeted agents—thereby broadening therapeutic choices—countries such as Armenia, Serbia, Montenegro, and Ukraine offer either limited access (covering only one drug) or no reimbursement at all. These discrepancies often mirror broader economic and structural challenges. Although the exact reasons for the absence of reimbursed CLL therapies in Armenia and Ukraine have not been officially disclosed and fall under the jurisdiction of the respective national health ministries, several likely contributing factors can be suggested. In Ukraine, the lack of reimbursement may be attributed to uncertainty surrounding the cost-effectiveness assessments conducted through local HTA processes. In Armenia, the historical absence of a clinical standard protocol for the management of CLL in adult patients likely delayed formal consideration of reimbursement pathways. Furthermore, countries such as Armenia, Serbia, Ukraine, and Montenegro—which represent the lowest GDP *per capita* group among those analyzed—may be perceived by pharmaceutical companies as less commercially attractive markets, potentially leading to delays in the submission of reimbursement applications. One noteworthy finding of our study is that a higher GDP *per capita* is positively correlated with broader coverage of novel therapies. In contrast, other examined variables (population size, life expectancy, the proportion of elderly individuals, healthcare spending)—aside from a borderline correlation with certain CLL epidemiological indicators, which implies that countries with a higher disease burden may be somewhat more inclined to reimburse a greater number of novel treatments—did not show a statistically significant relationship with reimbursement patterns. This suggests that economic capacity remains the primary determinant of whether and when these high-cost treatments are adopted, with other factors exerting only marginal or negligible influence.

This divergence in access is further highlighted by significant delays in reimbursement timelines, which may be driven by a combination of formal procedural requirements (such as the duration of HTA and reimbursement dossier assessments), strategic decisions by pharmaceutical companies regarding whether and when to initiate reimbursement processes, and the length of price negotiations with national authorities. While Israel, which does not require a formal HTA, consistently demonstrated the fastest reimbursement times for multiple drugs, other countries experienced significantly longer processes, sometimes extending to 4 years or more. These delays likely result from a combination of administrative inefficiencies, the lack of HTA adaptation to the specific challenges of rare diseases, and budget constraints. In many analyzed countries, HTA bodies do not fully consider unmet therapeutic needs—such as the lack of reimbursed targeted therapies—while often expressing skepticism toward indirect comparisons and struggling to conduct robust cost-effectiveness analyses in the absence of a standardized treatment approach. For example, the pivotal clinical trial for ibrutinib in the R/R CLL population (RESONATE) (Byrd et al., 2014) compared ibrutinib against ofatumumab, a therapy rarely considered a standard of care in analyzed countries for this indication. As a result, national HTA submissions had to use different comparators, including bendamustine, temsirolimus, chlorambucil, high-dose methylprednisolone + rituximab, best supportive care, or various immunochemotherapy regimens. This variability introduced uncertainty regarding ibrutinib’s relative benefit and cost-effectiveness, which may have contributed to delays in reimbursement decisions. Similarly, venetoclax monotherapy is primarily indicated after prior treatment with a BCRi, making access to ibrutinib or idelalisib a prerequisite for its use in accordance with its SmPC. For venetoclax + rituximab, the clinical trial comparator was bendamustine + rituximab (Seymour et al., 2018). However, since many countries already reimburse ibrutinib, indirect comparisons—often of limited reliability—became necessary, further prolonging the reimbursement process. On the other hand, second-generation BTKis, such as acalabrutinib and zanubrutinib, were reimbursed more rapidly in our study than ibrutinib. This expedited process likely reflects the fact that payers, having already approved an earlier BTKi for the same indication, faced fewer uncertainties when assessing subsequent agents within the same class. Since BTKis have comparable pricing and directly compete with one another, the financial impact of adding a new BTKi to reimbursement lists is often minimal. As a result, once a BTKi secures reimbursement, subsequent approvals within the same class typically encounter fewer barriers, resulting in shorter reimbursement timelines. Our study suggests that prolonged waiting times remain a significant barrier for first-in-class therapies that fulfill a major unmet medical need. In contrast, subsequent drugs within an established class appear to gain reimbursement approval more easily, despite potentially lower urgency. This discrepancy presents a challenge for patients who develop resistance or relapse and urgently require therapies with novel mechanisms of action. In such cases, timely access to innovative therapies can be crucial for survival outcomes and quality of life, highlighting the need for more flexible and expedited reimbursement pathways.

Reimbursement restrictions on approved indications further exacerbate access disparities in the analyzed region, adding another barrier alongside the limited number of reimbursed drugs and prolonged reimbursement timelines. Although some countries adhere to the full licensed labeling, many impose additional conditions related to mutational status (e.g., del17p or TP53) or timing of relapse. In some countries, access is limited to patients with particularly high-risk cytogenetics or those who fail multiple prior lines of therapy within a short timeframe. In other countries, restrictive administrative processes (e.g., treatment center designations, therapy monitoring programs, or mandated re-evaluations) limit where and how targeted agents can be prescribed. The rationale for such indication restrictions is not publicly disclosed and may stem from pricing negotiations with national health authorities or strategic decisions by pharmaceutical companies to seek reimbursement for a narrower patient population, thereby aiming to reduce the anticipated budget impact. While these measures can be economically justified considering budgetary constraints, they raise ethical concerns about equity of access and the principle of providing optimal care to all patients. The resulting patchwork of eligibility criteria often prevents clinicians from tailoring therapy to the individual clinical needs of their patients, further highlighting persistent inequities in oncology care across the region.

Another major challenge lies in the limited transparency of reimbursement policy and HTA processes, which often leaves the public and healthcare stakeholders uncertain about how coverage decisions are ultimately made. In many instances, publicly accessible data on the number of patients treated or the exact budget impact of newly approved therapies are simply unavailable, making direct cross-country comparisons difficult. The overall paucity of robust patient-level data points to a clear need for more transparent and standardized reporting on treatment uptake and outcomes to inform reimbursement decisions.

HTA processes themselves also vary widely. While most countries formally require HTA, the degree of transparency, methodological rigor, and WTP thresholds differ substantially. Some countries have clearly defined thresholds tied to GDP *per capita* or set at fixed monetary limits; others lack any explicit ceiling at all. Moreover, the absence of comparative clinical data—especially head-to-head trials in many cases—complicates cost-effectiveness evaluations, thus delaying or even preventing reimbursement. Cost-effectiveness results are also generally not disclosed to the public, making it difficult to assess the rationale behind reimbursement decisions and the transparency of the entire process. Risk-sharing agreements, confidential discounts, and managed entry programs might help reconcile concerns over financial feasibility, but their details remain largely undisclosed, limiting the ability of patients, clinicians, and the public to fully grasp how such deals affect coverage decisions.

From a policy perspective, these findings highlight the continuing tension between rapidly evolving oncology innovation and the slower pace of national reimbursement systems—especially in resource-constrained environments. Although CLL is classified as a rare disease, most countries have not implemented specialized orphan-drug policies to accelerate access to targeted therapies. In Armenia, for example, CLL is not even recognized as a rare disease and is covered solely under general healthcare financing regulations. Full reimbursement for chemotherapy is limited to vulnerable groups at approximately USD 750 per year, with non-vulnerable individuals receiving only half that amount, effectively placing targeted therapies out of reach. In other countries, national and geopolitical pressures—such as the war in Ukraine or administrative fragmentation in Bosnia and Herzegovina—further strain limited resources, underscoring how inadequate transparency can exacerbate disparities in patient access.

Overall, this study offers a comprehensive view of reimbursement patterns for R/R CLL therapies across CEE and Balkan countries, with additional insights from Armenia and Israel. This broad scope allows for more inclusive analyses, shedding light on the distinct policy environments that shape the funding and accessibility of novel therapies for R/R CLL within vastly different healthcare systems. Furthermore, the mixed-methods data collection strategy—merging quantitative survey findings with qualitative insights—enables a holistic view of real-world reimbursement environments. Finally, the article’s country-specific insights, covering aspects such as coverage pathways and eligibility criteria, offer valuable guidelines for policymakers and clinical stakeholders seeking to harmonize or reform reimbursement strategies in their respective contexts.

Nevertheless, the study also faces certain methodological limitations that may affect the depth or generalizability of the findings. First, although a cross-sectional mixed-methods approach using structured expert surveys is appropriate given the heterogeneity of national reimbursement systems, some data gaps may persist despite comprehensive efforts to ensure accuracy through publicly available databases and national sources. While this approach remains one of the few feasible strategies for conducting cross-national comparisons in this field, it inherently carries a risk of bias due to its reliance on expert opinion. Unfortunately, in this policy area, validated survey instruments and standardized frameworks for assessing access and reimbursement are still lacking. Second, we identified significant challenges related to data availability and quality, further complicated by inconsistent reporting practices across countries—particularly regarding real-world patient numbers, proposed prices, and confidential pricing agreements. Limited transparency in pricing negotiations remains a barrier, as list prices can differ significantly from the actual costs covered by payers under managed-entry agreements (MEAs). Due to the confidential nature of such arrangements, we were not able to assess the role of MEAs in shaping access. Third, the cross-sectional nature of the survey captures a snapshot of reimbursement at a single time point. Ongoing revisions in national pricing and HTA policies, as well as expansions or contractions of coverage, may alter the landscape of access. Finally, while the article includes basic correlation analyses, a more advanced statistical approach could allow for better control of confounding variables, thereby strengthening interpretations of how macroeconomic factors and policy frameworks influence reimbursement outcomes. However, this approach was limited by data availability and completeness, restricting the ability to explore potential direct causal relationships further.

Another potential limitation of this study lies in its scope. While it offers a detailed analysis of system-level determinants of access, it does not examine how variations in reimbursement translate into differences in clinical outcomes or survival across countries. Available real-world evidence from the region provides valuable, although limited, insights into clinical outcomes among patients with R/R CLL. In Romania, where reimbursement criteria align with the EMA-approved label, ibrutinib demonstrated an overall response rate (ORR) of 86% in 107 patients, with a median progression-free survival (PFS) of 50 months, outperforming outcomes reported in the pivotal RESONATE trial (Moldovianu et al., 2023). Similarly, a high ORR was observed in the Czech Republic (88.5%), although the median PFS was lower (40.5 months), closely mirroring the RESONATE results (Munir et al., 2019). This may reflect national reimbursement restrictions that exclude patients with late relapses (Špaček et al., 2023).

Comparable findings were reported by the Polish Adult Leukemia Group (PALG) (Soboń et al., 2023), which analyzed outcomes in 117 patients with R/R CLL treated with venetoclax–rituximab under the national reimbursement program. While the ORR among evaluable patients was 95.3% (86.3% in the overall cohort), the median PFS was only 36.97 months (Soboń et al., 2023)—substantially shorter than that reported in the pivotal MURANO trial (Kater et al., 2019). The authors attributed this discrepancy to the program’s restrictive eligibility criteria at the time, which limited access for patients with high-risk features such as early relapse, multiple prior therapies, or the presence of del17p/mTP53. These limitations likely reflected economic considerations aimed at prioritizing treatment for patients with the fewest available alternatives (Soboń et al., 2023). A Slovakian retrospective study of 43 patients with R/R CLL treated with venetoclax reported an even shorter median PFS of 31 months among those receiving the venetoclax–rituximab combination, while the median PFS in the monotherapy subgroup was not reached (Holasová et al., 2025). In Slovakia, access to the venetoclax–rituximab combination is restricted to patients with early relapse (≤18 months), whereas monotherapy is reimbursed without additional restrictions, consistent with the approved indication. These studies underscore the impact of reimbursement policies on treatment outcomes.

Another key insight from real-world evidence is the critical importance of securing reimbursement for multiple targeted therapies across distinct drug classes. Data from the Polish PALG registry highlight the poor prognosis of patients with R/R CLL who discontinue ibrutinib—regardless of the reason—with a median overall survival (OS) of only 2.0 months (Iskierka-Jażdżewska et al., 2019). This finding underscores the necessity of having alternative reimbursed treatment options for patients who are either intolerant or refractory to BTKis. Real-world data also raise important concerns about the lack of idelalisib reimbursement in many countries. While data from the Czech registry demonstrated significantly poorer outcomes with idelalisib–rituximab compared to ibrutinib (median PFS: 22.0 vs. 40.5 months; median OS: 37.7 vs. 54.4 months) (Špaček et al., 2023), findings from a Polish compassionate use program suggest more comparable effectiveness. Among 50 patients treated with idelalisib–rituximab, the estimated 12-month PFS and OS rates were 82.4% and 84.9%, respectively, with no significant differences in ORR or CR rate compared to ibrutinib-treated patients (Puła et al., 2018). These divergent outcomes reflect ongoing uncertainty regarding the real-world value of idelalisib and suggest that its systematic exclusion from reimbursement may not be fully justified.

While real-world clinical outcomes are partially available for some of the countries of interest, there remains a notable lack of region-specific data capturing the perspectives of patients and healthcare providers—particularly concerning delays in care, treatment discontinuation, affordability-related barriers, and unmet clinical needs. Although these aspects fall outside the scope of the current policy-focused study, they represent critical areas for future research. Survey-based studies involving both patients and healthcare professionals could help fill this gap by providing firsthand insights and complementing system-level findings with real-world care perspectives. In addition, there is a lack of published cost-effectiveness and budget impact analyses from academic or independent sources within the region, limiting the ability to contextualize reimbursement decisions in terms of economic value and affordability.

Future efforts should focus on improving regional collaboration and harmonizing HTA standards, potentially through joint assessments or shared best practices. The recent implementation of the Joint Clinical Assessment (JCA) framework under the EU HTA Regulation, effective as of 12 January 2025, for oncological drugs, represents a key advancement in this area (Fernandez et al., 2025). The JCA aims to streamline the clinical evaluation of new medicines at the EU level to reduce duplication of effort and enhance consistency in national decision-making (Fernandez et al., 2025). Although it does not apply retroactively to the therapies analyzed in this study, the framework may facilitate faster and more aligned reimbursement decisions for future treatments of R/R CLL. However, its actual impact remains uncertain, as implementation across member states is still evolving and national adaptation strategies may vary. Moreover, the JCA does not encompass economic evaluations, which will continue to be performed independently by each country, potentially limiting the extent of reimbursement harmonization. Facilitating real-world evidence generation—via registries or multi-country data-sharing platforms—could strengthen the evidence base on cost-effectiveness and inform more agile reimbursement decisions. Policymakers and stakeholders may also consider specialized funding avenues or risk-sharing frameworks tailored to rare hemato-oncological conditions, recognizing both the clinical urgency and the financial challenges involved. In this context, MEAs are gaining importance as tools to address uncertainty in both coverage and pricing decisions for new cancer therapies (Hofmarcher et al., 2024). Financial MEAs—such as confidential discounts, price-volume agreements, and expenditure caps—are more widely implemented due to their relative administrative simplicity (Hofmarcher et al., 2024). An alternative approach involves performance-based MEAs, which are particularly attractive for their ability to link reimbursement to real-world patient outcomes; however, their broader adoption remains limited by administrative complexity and insufficient IT infrastructure (Hofmarcher et al., 2024). Ultimately, enhancing transparency, equity, and evidence-based decision-making in reimbursement processes could help ensure that the promise of targeted therapies in R/R CLL is fully realized for patients across all European regions. Value frameworks such as the ESMO-Magnitude of Clinical Benefit Scale (ESMO-MCBS) and the WHO Model List of Essential Medicines (EML) aim to guide reimbursement decisions by prioritizing therapies that are clinically meaningful and highly effective (Hofmarcher et al., 2024). Although the targeted therapies assessed in our study were not evaluated within either framework, broader application of tools like the ESMO-MCBS and WHO EML could support more consistent, transparent, and equitable reimbursement decisions across Europe.

## 5 Conclusion

Significant disparities in the reimbursement of novel targeted therapies for R/R CLL persist across the analyzed group of CEE countries, the Balkans, Armenia, and Israel. Access to innovative treatments was highly variable, with the number of reimbursed therapies ranging from none (Ukraine, Armenia) to five (Czech Republic), and reimbursement delays spanning from very short in Israel to extremely long in Serbia. Access to reimbursed therapies was positively associated with countries’ economic status, as reflected by GDP *per capita*, while no significant associations between the number of reimbursed drugs and reimbursement delays were observed with other macroeconomic, demographic, or epidemiological factors. Although most countries formally require HTA evaluations (with exceptions including Serbia, Armenia, Bosnia and Herzegovina, Israel, and Croatia), the transparency, methodological rigor, duration of the process, and WTP thresholds vary substantially across the region. Key barriers to equitable access include administrative inefficiencies, insufficient adaptation of HTA frameworks to the specific needs of rare diseases, and persistent budgetary constraints.

## Data Availability

All data supporting the results of this study are presented in the article and in the Supplementary Material. Additional information is available from the corresponding author upon request.
