# Supplementary material for: EUROCOVER-CLL: Reimbursement and accessibility of new treatments in relapsed/refractory chronic lymphocytic leukemia
Source: Front Pharmacol. 2025 Sep 2;16:1629465. doi: 10.3389/fphar.2025.1629465 (PMC12436112; doi:10.3389/fphar.2025.1629465)
Supplement: Supplementary file 1 [file Supplementaryfile1.docx]

Supplementary Material

# Supplementary Data

**Supplementary File 1**

**PART 1:** Reimbursement Access to CLL therapies: This part focuses on gathering information about the availability and accessibility of reimbursement for R/R CLL therapies in your country. It includes questions about reimbursement indications, target populations, duration, and costs. It contains 13 specific questions regarding each therapy reimbursed in your country.

**Which therapies are reimbursed in relapsed/refractory chronic lymphocytic leukemia in your country?**

Relapsed/refractory concerns patients previously treated with at least one systemic therapy (chemotherapy, immune therapy, targeted therapy)

*Select several answers*

|  |  |
| --- | --- |
| 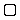 | Acalabrutinib |
| 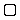 | Bendamustine+rituximab |
| 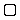 | Duvelisib |
| 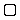 | Ibrutinib |
| 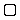 | Ibrutinib+bendamustine+rituximab |
| 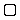 | Idelalisib+rituximab |
| 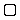 | Venetoclax |
| 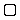 | Venetoclax+rituximab |
| 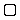 | Zanubrutinib |
| 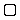 | None of the above |

**1. Please provide the exact reimbursement indication of [drug] in relapsed/refractory CLL patients (current)**

Examples: "2nd line treatment and beyond for CLL" or "Only in patients without 17p deletion or TP53 mutation, ECOG 0-1, age 60 years"

| _ _ _ _ _ _ _ _ _ _ _ _ _ _ _ _ _ _ _ _ _ _ _ _ |
| --- |

**2. Since when has [drug] been reimbursed?**

Please provide the initial reimbursement date for R/R CLL patients in format DD-MM-YYYY.

| _ _ _ _ _ _ _ _ _ _ _ _ _ _ _ _ _ _ _ _ _ _ _ _ |
| --- |

**3. Has the reimbursement indication for [drug] changed after its initial reimbursement decision?**

Please provide additional information about previous indications.

*Select only one answer*

|  |  |
| --- | --- |
| 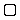 | No, it has remained the same |
| 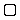 | Yes, it has been narrowed (please provide details):  _ _ _ _ _ _ _ _ _ _ _ _ _ _ _ _ _ _ _ _ _ _ _ _ _ _ _ _ |
| 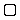 | Yes, it has been expanded (please provide details):  _ _ _ _ _ _ _ _ _ _ _ _ _ _ _ _ _ _ _ _ _ _ _ _ _ _ _ _ |
| 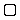 | Other (please specify):  _ _ _ _ _ _ _ _ _ _ _ _ _ _ _ _ _ _ _ _ _ _ _ _ _ _ _ _ |

**4. Was the current reimbursement decision for [drug] granted indefinitely or temporarily?**

Choose which one applies

*Select only one answer*

|  |  |
| --- | --- |
| 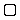 | Indefinitely (no specific end date) |
| 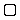 | Temporarily (please provide end date):  _ _ _ _ _ _ _ _ _ _ _ _ _ _ _ _ _ _ _ _ _ _ _ _ _ _ _ _ |

**5. What is the size of the target population for which [drug] is reimbursed?**

Please provide an exact or estimated number of patients treated with the therapy by each year (if possible, for R/R CLL-specific indications). For example: 1) Exact numbers: 2016: 21, 2017: 56, 2018: 112, 2019: 451, 2020: 510, 2021: 512 2) Estimated numbers: 2016: 100, 2017: 450 etc.

| _ _ _ _ _ _ _ _ _ _ _ _ _ _ _ _ _ _ _ _ _ _ _ _    _ _ _ _ _ _ _ _ _ _ _ _ _ _ _ _ _ _ _ _ _ _ _ _    _ _ _ _ _ _ _ _ _ _ _ _ _ _ _ _ _ _ _ _ _ _ _ _ |
| --- |

**6. Is [drug] eligible for reimbursement in inpatient care (hospitals) or outpatient care (ambulatory)?**

Select all that apply

*Select several answers*

|  |  |
| --- | --- |
| 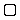 | Inpatient care |
| 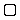 | Outpatient care |
| 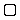 | Other (please specify):  _ _ _ _ _ _ _ _ _ _ _ _ _ _ _ _ _ _ _ _ _ _ _ _ _ _ _ _ |

**7. Is there a specific reimbursement program or scheme through which [drug] is reimbursed?**

Choose which applies the best

*Select only one answer*

|  |  |
| --- | --- |
| 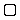 | Yes, there is a specific program or scheme (if so, please provide details about the program or scheme):  _ _ _ _ _ _ _ _ _ _ _ _ _ _ _ _ _ _ _ _ _ _ _ _ _ _ _ _ |
| 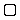 | No, there is no specific program or scheme (If so, please provide how the therapy is reimbursed (e.g., national health insurance, guaranteed health service):  _ _ _ _ _ _ _ _ _ _ _ _ _ _ _ _ _ _ _ _ _ _ _ _ _ _ _ _ |

**8. What is the level of reimbursement for [drug] in your country?**

Choose which applies the best

*Select only one answer*

|  |  |
| --- | --- |
| 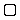 | Full reimbursement (100% coverage) |
| 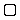 | Partial reimbursement (please provide x% coverage):  _ _ _ _ _ _ _ _ _ _ _ _ _ _ _ _ _ _ _ _ _ _ _ _ _ _ _ _ |
| 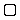 | Fixed reimbursement amount (please specify monetary value):  _ _ _ _ _ _ _ _ _ _ _ _ _ _ _ _ _ _ _ _ _ _ _ _ _ _ _ _ |
| 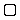 | Other (please specify):  _ _ _ _ _ _ _ _ _ _ _ _ _ _ _ _ _ _ _ _ _ _ _ _ _ _ _ _ |

**9. What is the current official price of [drug] in your country?**

Please specify the price or provide information on how the pricing is determined

| _ _ _ _ _ _ _ _ _ _ _ _ _ _ _ _ _ _ _ _ _ _ _ _ |
| --- |

**10. How have the official prices of [drug] changed since the first reimbursement decision for this therapy?**

Select which applies

*Select only one answer*

|  |  |
| --- | --- |
| 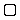 | Prices have increased over time (if so, please provide details):  _ _ _ _ _ _ _ _ _ _ _ _ _ _ _ _ _ _ _ _ _ _ _ _ _ _ _ _ |
| 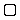 | Prices have decreased over time (if so, please provide details):  _ _ _ _ _ _ _ _ _ _ _ _ _ _ _ _ _ _ _ _ _ _ _ _ _ _ _ _ |
| 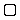 | Prices have remained relatively stable over time |
| 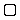 | Other (please specify):  _ _ _ _ _ _ _ _ _ _ _ _ _ _ _ _ _ _ _ _ _ _ _ _ _ _ _ _ |

**11. What are the reimbursement costs associated with [drug] per year?**

Please provide exact or estimated costs

| _ _ _ _ _ _ _ _ _ _ _ _ _ _ _ _ _ _ _ _ _ _ _ _ |
| --- |

**12. In your country, are there any out-of-pocket costs or co-payments that patients need to bear for [drug]?**

Choose which applies the best

*Select only one answer*

|  |  |
| --- | --- |
| 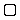 | Yes, there are out-of-pocket costs or co-payments (if so, please provide estimated costs per patient):  _ _ _ _ _ _ _ _ _ _ _ _ _ _ _ _ _ _ _ _ _ _ _ _ _ _ _ _ |
| 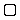 | No, there are no out-of-pocket costs or co-payments |

**13. Are there any additional limitations or conditions related to the reimbursement of [drug]?**

Please provide any additional limitations regarding the reimbursement of the therapy in R/R CLL

| _ _ _ _ _ _ _ _ _ _ _ _ _ _ _ _ _ _ _ _ _ _ _ _ |
| --- |

**PART 2:** Health Technology Assessment (HTA) Evaluation: This part assesses whether HTA evaluation for the assessed therapies was conducted and examines publicly available information regarding cost-effectiveness indicators for the treatment. It includes questions about the outcome of the HTA, clinical and economic results, comparators for the HTA evaluation, and risk-sharing agreements. It contains 10 specific questions regarding each therapy assessed in your country.

**For which therapies has HTA evaluation been conducted in relapsed/refractory chronic lymphocytic leukemia in your country?**

Relapsed/refractory concerns patients previously treated with at least one systemic therapy (chemotherapy, immune therapy, targeted therapy)

*Select several answers*

|  |  |
| --- | --- |
| 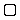 | Acalabrutinib |
| 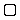 | Bendamustine+rituximab |
| 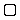 | Duvelisib |
| 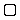 | Ibrutinib |
| 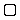 | Ibrutinib+bendamustine+rituximab |
| 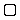 | Idelalisib+rituximab |
| 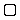 | Venetoclax |
| 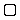 | Venetoclax+rituximab |
| 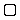 | Zanubrutinib |
| 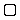 | None of the above |

**1. What comparators were used in the HTA evaluation for [drug] in R/R indication?**

Please provide comparators for all indications. For example: ofatumumab for all patients, venetoclax+ibrutiinib for del17p/mTP53+ etc. Note: Comparators in your responses can include other treatments not previously mentioned in this survey, like ofatumumab, rituximab, chlorambucil, etc.

| _ _ _ _ _ _ _ _ _ _ _ _ _ _ _ _ _ _ _ _ _ _ _ _ |
| --- |

**2. Based on the HTA evaluation, was the comparison of treatment effect conducted through direct or indirect comparisons?**

For example: direct - ofatumumab; indirect - MAIC - rituximab; indirect - naïve: venetoclax+ibrutinib Note: Comparators in your responses can include other treatments not previously mentioned in this survey, like ofatumumab, rituximab, chlorambucil, etc.

*Select several answers*

|  |  |
| --- | --- |
| 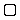 | Direct comparison (specify comparator(s)):  _ _ _ _ _ _ _ _ _ _ _ _ _ _ _ _ _ _ _ _ _ _ _ _ _ _ _ _ |
| 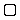 | Indirect comparison – network meta-analysis (specify comparator(s)):  _ _ _ _ _ _ _ _ _ _ _ _ _ _ _ _ _ _ _ _ _ _ _ _ _ _ _ _ |
| 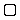 | Indirect comparison – matching-adjusted indirect comparison (specify (comparator(s)):  _ _ _ _ _ _ _ _ _ _ _ _ _ _ _ _ _ _ _ _ _ _ _ _ _ _ _ _ |
| 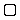 | Indirect – naïve comparison (specify (comparator(s)):  _ _ _ _ _ _ _ _ _ _ _ _ _ _ _ _ _ _ _ _ _ _ _ _ _ _ _ _ |
| 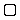 | Indirect comparison – adjusted comparison (Bucher) (specify (comparator(s)):  _ _ _ _ _ _ _ _ _ _ _ _ _ _ _ _ _ _ _ _ _ _ _ _ _ _ _ _ |

**3. Did the HTA evaluation demonstrate the clinical benefit (e.g., higher efficacy or better safety profile) of [drug] over the comparator(s)?**

For example: Yes, clinical superiority was demonstrated: ofatumumab, No clinical superiority was not demonstrated: chlorambucil+rituximab Note: Comparators in your responses can include other treatments not previously mentioned in this survey, like ofatumumab, rituximab, chlorambucil, etc.

*Select several answers*

|  |  |
| --- | --- |
| 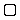 | Yes, clinical superiority was demonstrated (specify comparator(s)):  _ _ _ _ _ _ _ _ _ _ _ _ _ _ _ _ _ _ _ _ _ _ _ _ _ _ _ _ |
| 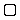 | No, clinical superiority was not demonstrated specify comparator(s)):  _ _ _ _ _ _ _ _ _ _ _ _ _ _ _ _ _ _ _ _ _ _ _ _ _ _ _ _ |
| 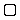 | Not applicable (HTA evaluation did not assess clinical superiority) |
| 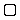 | Clinical benefit remains uncertain (specify (comparator(s)):  _ _ _ _ _ _ _ _ _ _ _ _ _ _ _ _ _ _ _ _ _ _ _ _ _ _ _ _ |

**4. What type of economic analysis was conducted as a part of HTA evaluation?**

For example: CEA: ofatumumab, CMA: chlorambucil+rituximab Note: Comparators in your responses can include other treatments not previously mentioned in this survey, like ofatumumab, rituximab, chlorambucil, etc.

*Select several answers*

|  |  |
| --- | --- |
| 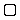 | Cost-effectiveness analysis (CEA) (specify comparator(s)):  _ _ _ _ _ _ _ _ _ _ _ _ _ _ _ _ _ _ _ _ _ _ _ _ _ _ _ _ |
| 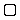 | Cost-utility analysis (CUA)(specify comparator(s)):  _ _ _ _ _ _ _ _ _ _ _ _ _ _ _ _ _ _ _ _ _ _ _ _ _ _ _ _ |
| 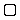 | CEA and CUA (specify comparator(s)):  _ _ _ _ _ _ _ _ _ _ _ _ _ _ _ _ _ _ _ _ _ _ _ _ _ _ _ _ |
| 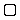 | Cost-consequence analysis (CCA) (specify comparator(s)):  _ _ _ _ _ _ _ _ _ _ _ _ _ _ _ _ _ _ _ _ _ _ _ _ _ _ _ _ |
| 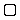 | Cost minimization analysis (CMA) (specify comparator(s)):  _ _ _ _ _ _ _ _ _ _ _ _ _ _ _ _ _ _ _ _ _ _ _ _ _ _ _ _ |
| 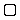 | Other (please specify details and comparator(s)):  _ _ _ _ _ _ _ _ _ _ _ _ _ _ _ _ _ _ _ _ _ _ _ _ _ _ _ _ |
| 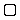 | None |

**5. Was cost-effectiveness or cost-utility demonstrated in the HTA evaluation for the [drug] in R/R CLL?**

For example: Yes, cost-effectiveness or cost-utility was demonstrated: ofatumumab, No, cost-effectiveness or cost-utility was not demonstrated: chlorambucil+rituximab Note: Comparators in your responses can include other treatments not previously mentioned in this survey, like ofatumumab, rituximab, chlorambucil, etc.

*Select several answers*

|  |  |
| --- | --- |
| 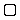 | Yes, cost-effectiveness or cost-utility was demonstrated (please specify comparator(s)) |
| 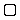 | No, cost-effectiveness or cost-utility was not demonstrated (please specify comparator(s)) |
| 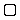 | Not applicable (HTA evaluation did not assess cost-effectiveness or cost-utility) |
| 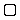 | Unknown (information not publicly available) |

**6. If applicable, what was the Incremental Cost-Effectiveness Ratio (ICER) or Incremental Cost-Utility Ratio (ICUR) reported in the HTA evaluation?**

For example: ICER: 25 000 EUR/LY - ofatumumab, ICUR: 85 000 EUR/QALY - chlorambucil+rituximab Note: Comparators in your responses can include other treatments not previously mentioned in this survey, like ofatumumab, rituximab, chlorambucil, etc.

*Select several answers*

|  |  |
| --- | --- |
| 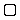 | ICER (please specify comparator(s) and ICER value(s)):  _ _ _ _ _ _ _ _ _ _ _ _ _ _ _ _ _ _ _ _ _ _ _ _ _ _ _ _ |
| 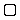 | ICUR (please specify comparator(s) and ICUR value(s)):  _ _ _ _ _ _ _ _ _ _ _ _ _ _ _ _ _ _ _ _ _ _ _ _ _ _ _ _ |
| 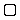 | Information not publicly available |
| 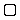 | Not applicable (HTA evaluation did not assess cost-effectiveness or cost-utility) |

**7. How much time has passed between publishing the HTA recommendation and including [drug] in the reimbursement list?**

*Select only one answer*

|  |  |
| --- | --- |
| 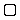 | Less than 6 months |
| 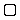 | 6 months to 1 year |
| 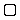 | 1 to 2 years |
| 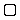 | More than 2 years |
| 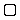 | Not reimbursed yet |
| 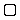 | Information not publicly available |

**8. Has a risk-sharing scheme or similar risk-sharing instruments been implemented in the reimbursement process?**

*Select only one answer*

|  |  |
| --- | --- |
| 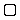 | Yes, a risk-sharing scheme has been implemented |
| 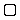 | No, a risk-sharing scheme has not been implemented |
| 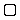 | No information |

**9. Is the risk-sharing agreement publicly available?**

*Select only one answer*

|  |  |
| --- | --- |
| 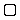 | Yes, the risk-sharing agreement is publicly available. |
| 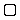 | No, the risk-sharing agreement is not publicly available. |
| 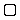 | Not applicable (a risk-sharing instrument has not been implemented) |

**10. What are the details of the risk-sharing agreement, if publicly available?**

Skip the question if no information is publicly available.

| _ _ _ _ _ _ _ _ _ _ _ _ _ _ _ _ _ _ _ _ _ _ _ _    _ _ _ _ _ _ _ _ _ _ _ _ _ _ _ _ _ _ _ _ _ _ _ _    _ _ _ _ _ _ _ _ _ _ _ _ _ _ _ _ _ _ _ _ _ _ _ _ |
| --- |

**PART 3:** General Reimbursement Policy: This part explores the specific reimbursement policy in your country. It includes questions regarding the requirements for reimbursement, the necessity of HTA assessments for obtaining reimbursement, the willingness-to-pay thresholds, the duration of HTA evaluations, and the submission timeline for HTA applications. It contains 10 general questions.

**1. In your country, what is the overall reimbursement policy for hematooncological therapies, including those for CLL?**

Select which applies. If possible, provide details.

*Select only one answer*

|  |  |
| --- | --- |
| 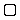 | There is a specific reimbursement policy for oncology and hematology therapies (if possible, provide additional details):  _ _ _ _ _ _ _ _ _ _ _ _ _ _ _ _ _ _ _ _ _ _ _ _ _ _ _ _ |
| 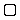 | There is a general reimbursement policy that covers various therapeutic areas, including oncology and hematology |

**2. Are there any specific criteria or requirements for reimbursement of hematooncological therapies in your country?**

Select all that apply

*Select several answers*

|  |  |
| --- | --- |
| 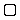 | Efficacy and safety criteria |
| 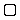 | HTA evaluation |
| 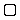 | Specific eligibility criteria for patients |
| 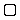 | Other (please specify):  _ _ _ _ _ _ _ _ _ _ _ _ _ _ _ _ _ _ _ _ _ _ _ _ _ _ _ _ |

**3. How does the reimbursement policy in your country consider the availability of innovative or targeted therapies for oncology and hematology conditions, including CLL?**

Select which applies

*Select only one answer*

|  |  |
| --- | --- |
| 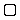 | There are specific provisions or programs to facilitate access to innovative or targeted therapies (if possible, please provide details):  _ _ _ _ _ _ _ _ _ _ _ _ _ _ _ _ _ _ _ _ _ _ _ _ _ _ _ _ |
| 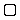 | Access to innovative or targeted therapies is determined on a case-by-case basis |
| 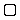 | There are no specific considerations for innovative or targeted therapies |

**4. Are there additional reimbursement considerations or pathways specifically designed for rare or orphan oncology and hematology therapies?**

*Select only one answer*

|  |  |
| --- | --- |
| 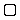 | Yes, there are specific reimbursement considerations for rare or orphan therapies (if so, please specify):  _ _ _ _ _ _ _ _ _ _ _ _ _ _ _ _ _ _ _ _ _ _ _ _ _ _ _ _ |
| 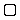 | No, there are no specific reimbursement considerations for rare or orphan therapies |

**5. If different reimbursement rules apply to rare diseases, have they been implemented in the case of CLL?**

| _ _ _ _ _ _ _ _ _ _ _ _ _ _ _ _ _ _ _ _ _ _ _ _    _ _ _ _ _ _ _ _ _ _ _ _ _ _ _ _ _ _ _ _ _ _ _ _  _ _ _ _ _ _ _ _ _ _ _ _ _ _ _ _ _ _ _ _ _ _ _ _ |
| --- |

**6. Is an HTA evaluation required in your country to obtain reimbursement?**

Please select which applies

*Select only one answer*

|  |  |
| --- | --- |
| 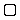 | Yes, an HTA evaluation is generally required for obtaining reimbursement |
| 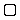 | Yes, an HTA evaluation is required, but there are exceptions (please specify):  _ _ _ _ _ _ _ _ _ _ _ _ _ _ _ _ _ _ _ _ _ _ _ _ _ _ _ _ |
| 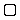 | No, an HTA evaluation is not required for obtaining reimbursement |
| 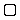 | Not applicable (HTA evaluation does not apply to the reimbursement process in my country) |

**7. How long did the HTA evaluation process typically last, from initiation to the release of the final evaluation report?**

Please provide an exact or estimated time in months

| _ _ _ _ _ _ _ _ _ _ _ _ _ _ _ _ _ _ _ _ _ _ _ _ |
| --- |

**8. Is there a specific willingness-to-pay (WTP) threshold used in your country to determine the cost-effectiveness of therapies for reimbursement decisions?**

*Select only one answer*

|  |  |
| --- | --- |
| 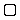 | Yes, there is a specific WTP threshold (if so, please specify):  _ _ _ _ _ _ _ _ _ _ _ _ _ _ _ _ _ _ _ _ _ _ _ _ _ _ _ _ |
| 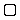 | No, there is no specific WTP threshold (WTP threshold does not apply to reimbursement decisions in my country) |

**9. What are the overall reimbursement expenditures for pharmacotherapy in your country?**

Please provide an exact or estimated number

| _ _ _ _ _ _ _ _ _ _ _ _ _ _ _ _ _ _ _ _ _ _ _ _    _ _ _ _ _ _ _ _ _ _ _ _ _ _ _ _ _ _ _ _ _ _ _ _ |
| --- |

**10. How frequently do the lists of reimbursed medications change in your country?**

Please select which applies

*Select only one answer*

|  |  |
| --- | --- |
| 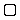 | Very frequently (monthly or every two months) |
| 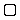 | Frequently (every three or four months) |
| 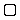 | Rarely (once or twice per year) |
| 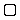 | Occasionally (less often than once a year) |

**PART 4:** General Reimbursement Situation: This part focuses on your overall perspective of the reimbursement landscape for CLL medications, including the compliance between clinical guidelines and reimbursement policies, the availability of patient support programs, and your opinion regarding any challenges or difficulties encountered in the reimbursement process. It contains 10 general questions.

**1. Do you consider the current reimbursement system fair and accessible for patients being treated for chronic lymphocytic leukemia?**

*Select only one answer*

|  |  |
| --- | --- |
| 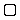 | Yes, the current reimbursement system is fair and accessible |
| 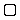 | No, the current reimbursement system is not fair and accessible (If so, please specify why):  _ _ _ _ _ _ _ _ _ _ _ _ _ _ _ _ _ _ _ _ _ _ _ _ _ _ _ _ |
|  | Unsure / I don't know |

**2. Are there any differences in reimbursement policies or coverage for different subtypes or stages of chronic lymphocytic leukemia? If yes, what factors could potentially contribute to unequal access to therapy?**

Please select all that applies

*Select several answers*

|  |  |
| --- | --- |
|  | Variation in clinical guidelines and treatment recommendations |
|  | Differences in the availability of specific therapies or targeted agents for different CLL subtypes or stages |
|  | Disparities in evidence or clinical data supporting the effectiveness of treatments for specific CLL subtypes or stages |
|  | Budgetary constraints or limitations in healthcare funding |
|  | Variation in the interpretation and implementation of reimbursement criteria by different regions or authorities |
|  | Challenges in accessing or obtaining diagnostic tests or biomarker assessments for precise CLL subtype or stage determination |
|  | Other (please specify):  _ _ _ _ _ _ _ _ _ _ _ _ _ _ _ _ _ _ _ _ _ _ _ _ _ _ _ _ |
|  | Not applicable (there are no differences in reimbursement policies or coverage for different subtypes or stages of CLL) |

**3. Do you have any suggestions or comments for improving the reimbursement process for chronic lymphocytic leukemia medications in your country?**

Please select which applies

*Select only one answer*

|  |  |
| --- | --- |
|  | Yes, I have suggestions for improving the reimbursement process (if so, please specify):  _ _ _ _ _ _ _ _ _ _ _ _ _ _ _ _ _ _ _ _ _ _ _ _ _ _ _ _ |
|  | No, I do not have any suggestions or comments |

**4. Have you noticed any challenges or barriers in accessing the reimbursement therapies for chronic lymphocytic leukemia (e.g., lack of the reimbursement component in combinational therapies, lack of access to molecular laboratory testing to confirm patient’s eligibility for reimbursement)**

Please select which applies

*Select only one answer*

|  |  |
| --- | --- |
|  | Yes, there are challenges or barriers in accessing reimbursement therapies (if so, please specify):  _ _ _ _ _ _ _ _ _ _ _ _ _ _ _ _ _ _ _ _ _ _ _ _ _ _ _ _ |
|  | No, there are no significant challenges or barriers |
|  | Unsure / I don't know |

**5. Are there any specific national guidelines for clinicians regarding CLL?**

Please select which applies

*Select only one answer*

|  |  |
| --- | --- |
|  | Yes, there are specific national guidelines for clinicians (if so, please provide the reference):  _ _ _ _ _ _ _ _ _ _ _ _ _ _ _ _ _ _ _ _ _ _ _ _ _ _ _ _ |
|  | No, there are no specific national guidelines |

**6. Is the current reimbursement practice compliant with the therapies recommended by national guidelines for CLL?**

Please select which applies

*Select only one answer*

|  |  |
| --- | --- |
|  | Yes, the current reimbursement practice is compliant with the recommended therapies |
|  | No, the current reimbursement practice does not align with the recommended therapies (if so, please specify where discrepancies occur):  _ _ _ _ _ _ _ _ _ _ _ _ _ _ _ _ _ _ _ _ _ _ _ _ _ _ _ _ |
|  | Not applicable (there are no national guidelines for CLL) |

**7. Are there any patient support programs or financial assistance options for individuals which are not currently covered by reimbursement?**

*Select only one answer*

|  |  |
| --- | --- |
|  | Yes, there are patient support programs or financial assistance options available (if so, please provide details):  _ _ _ _ _ _ _ _ _ _ _ _ _ _ _ _ _ _ _ _ _ _ _ _ _ _ _ _ |
|  | No, there are no patient support programs or financial assistance options |
|  | Unsure / I don't know |

**8. Have you noticed any delays or difficulties in the reimbursement process for any CLL therapy?**

*Select only one answer*

|  |  |
| --- | --- |
|  | Yes, there have been delays or difficulties in the reimbursement process (if so, please provide details):  _ _ _ _ _ _ _ _ _ _ _ _ _ _ _ _ _ _ _ _ _ _ _ _ _ _ _ _ |
|  | No, there have been no significant delays or difficulties |
|  | Unsure / I don't know |

**9. Are any patient advocacy groups or organizations actively advocating for improved reimbursement policies for CLL?**

*Select only one answer*

|  |  |
| --- | --- |
|  | Yes, there are patient advocacy groups or organizations actively involved (if so, please provide details):  _ _ _ _ _ _ _ _ _ _ _ _ _ _ _ _ _ _ _ _ _ _ _ _ _ _ _ _ |
|  | No, there are no patient advocacy groups or organizations involved |
|  | Unsure / I don't know |

**10. Are there any alternative treatments or therapies that are not reimbursed but could be beneficial for patients with CLL?**

*Select only one answer*

|  |  |
| --- | --- |
|  | Yes, there are alternative treatments or therapies that could be beneficial (if so, please provide details about the treatment and the population):  _ _ _ _ _ _ _ _ _ _ _ _ _ _ _ _ _ _ _ _ _ _ _ _ _ _ _ _ |
|  | No, all beneficial treatments or therapies are currently reimbursed |
|  | Unsure / I don't know |

**Supplementary File 2**

**Data sources**

**Armenia**: 1. Frontiers in Oncology. (2021). Armenia’s reimbursement policies for oncology. Retrieved from <https://www.frontiersin.org/journals/oncology/articles/10.3389/fonc.2021.782581/full>; 2. EVN Report. (2021). Armenia's plan for universal health insurance. Retrieved from https://evnreport.com/lawsociety/ armenias-plan-for-universal-health-insurance/; 3. ARKA News. (2023). State subsidies for chemotherapy to increase significantly, says minister. Retrieved from <https://arka.am/en/news/society/state_subsidies_for_chemotherapy_to_increase_significantly_minister/>; 4. Journal of Thoracic Oncology. (2022). Analysis of targeted oncology therapies. Retrieved from <https://www.jto.org/article/S1556-0864%2822%2901991-8/fulltext>; 5. PPRI. (2023). Pharmaceutical pricing and reimbursement information: Armenia health system review. Retrieved from <https://ppri.goeg.at/sites/ppri.goeg.at/files/inline-files/Armenia.pdf>; 6. Ministry of Health, Armenia. (2021). Armenian eHealth strategy and action plan (2021-2023). Retrieved from <https://www.moh.am/uploads/eHealth%20Strategy_ENG.pdf>; 7. Scientific Centre of Drug and Medical Technology Expertise. (2023). Register of medicinal products in Armenia. Retrieved from <https://pharm.am>;8.Ministry of Economy, Armenia. (2024). Economic developments and pharmaceutical regulations. Retrieved from <https://mineconomy.am/en/page/3046>; 9. World Health Organization. (2015). Health technology assessment country profile: Armenia. Retrieved from <https://www.who.int/publications/m/item/health-technology-assessment-country-profile-armenia>; 10.World Health Organization. (2022). Health systems in action: Armenia. Retrieved from https://eurohealthobservatory.who.int/countries/armenia; 11.The Max Foundation. (2024). First doses of Brukinsa provided to patients with CLL in Armenia. Retrieved from <https://themaxfoundation.org/news/first-doses-of-brukinsa-provided-to-patients-with-chronic-lymphocytic-leukemiain-low-and-middle-income-countries/>; 12.EFPIA. (2020). Improving time to patient access to innovative oncology therapies in Europe. Retrieved from <https://www.efpia.eu/media/578013/every-day-counts.pdf>; 13. MacroTrends. (2021). Armenia healthcare spending data (2000-2024). Retrieved from <https://www.macrotrends.net/global-metrics/countries/ARM/armenia/healthcare-spending>; 14. Panorama Armenia. (2023). Armenian government plans 14% increase in health spending in 2024. Retrieved from <https://www.panorama.am/en/news/2023/11/06/health-spending/2921848>; 15. ArPharMa. (2024). Reimbursement updates and pharmaceutical market analysis in Armenia. Retrieved from <https://arpharma.am/en/2024/07/09/reimbursement-of-50-of-the-costs-of-conducting-an-examination-forregistration-and-re-registration-of-medicines-in-the-amount-of-120-million-drams-4/>; 16. Grove Online. (2024). Market access services in Armenia. Retrieved from <https://groveonline.com/country/market-access-services-in-armenia/>; 17. SpringerLink. (2023). HTA criteria and their evaluations. Retrieved from <https://link.springer.com/article/10.1007/s10238-023-01007-2>; 18. Value in Health Journal. (2023). Adaptive health technology assessment for reimbursement decisions. Retrieved from <https://www.valueinhealthjournal.com>; 19. World Bank. (2024). Technical support for universal health coverage in Armenia. Retrieved from https://documents1.worldbank.org/curated/en/099240012202120838/pdf/P171735080ea4708b0ad0f04cf87948b404

**Bosnia and Herzegovina:** 1. <http://www.fmoh.gov.ba/>; 2. <https://www.vladars.net/sr-SP-Cyrl/Vlada/Ministarstva/MZSZ/Pages/default.aspx>; 3. <https://mcp.gov.ba>; 4. <https://almbih.gov.ba/>;

**Bulgaria:** 1. <https://portal.ncpr.bg/registers/pages/register/archive.xhtml>; 2. <https://www.nhif.bg/bg>

**Croatia:** 1. <https://zdravlje.gov.hr/>; <https://zdravlje.gov.hr/o-ministarstvu/djelokrug-1297/lijekovi-i-medicinski-proizvodi/1349>; 2. <https://www.halmed.hr/>; 3. <https://www.halmed.hr/Lijekovi/Informacije-o-lijekovima/Lijekovi-za-lijecenje-rijetkih-i-teskih-bolesti/>; 4. <https://www.halmed.hr/Lijekovi/Informacije-o-lijekovima/Bioloski-i-bioslicni-lijekovi/>; 5. <https://hzzo.hr/>; 6. <https://hzzo.hr/zdravstvena-zastita/lijekovi/objavljene-liste-lijekova>

**Czech Republic:** 1. <https://www.sukl.cz>

**Estonia:** 1. <https://www.tervisekassa.ee/ravimikomisjoni-koosolekute-paevakorrad-ja-protokollid>; 2. <https://www.tervisekassa.ee/partnerile/ravimitest/soodusravimid>; 3. <https://www.riigiteataja.ee/akt/123122017021>

**Hungary:** 1. [https://www.neak.gov.hu/felso_menu/szakmai_oldalak/gyogyszer_segedeszkoz_gyogyfurdo_tamogatas/egeszsegugyi_vallalkozasoknak/gyartok_forgalomba_hozok/KERELEM_IND_ELJ_GYOGYSZ_TAPSZ 2](https://www.neak.gov.hu/felso_menu/szakmai_oldalak/gyogyszer_segedeszkoz_gyogyfurdo_tamogatas/egeszsegugyi_vallalkozasoknak/gyartok_forgalomba_hozok/KERELEM_IND_ELJ_GYOGYSZ_TAPSZ%202); <https://www.neak.gov.hu/felso_menu/szakmai_oldalak/gyogyszer_segedeszkoz_gyogyfurdo_tamogatas/egeszsegugyi_vallalkozasoknak/gyartok_forgalomba_hozok/tam_vol>; 3. <http://www.neak.gov.hu/felso_menu/szakmai_oldalak/gyogyszer_segedeszkoz_gyogyfurdo_tamogatas/egeszsegugyi_vallalkozasoknak/pupha>; 4. <https://njt.hu/jogszabaly/1993-9-20-3D>; 5. <https://ogyei.gov.hu/dynamic/NNGYK_TeF_Modszertani_Ajanlasok_20230928.pdf>

**Israel**: 1. Israel MOH website (In Hebrew) [https://www.gov.il/he/departments/units/vsal-committee-unit#1](https://www.gov.il/he/departments/units/vsal-committee-unit#1)**Poland:** 1. <https://mz.gov.pl>; 2. <https://bip.aotm.gov.pl/>; 3. <https://ikarpro.pl/pl>

**Romania:** 1. <https://www.anm.ro/medicamente-de-uz-uman/evaluare-tehnologii-medicale/rapoarte-de-evaluare-a-tehnologiilor-medicale/> – HTA body site with all HTA reports; 2. <https://cnas.ro/protocoale-terapeutice/> – the prescription protocols for all medicines reimbursed in Romania; 3. <https://www.anm.ro/medicamente-de-uz-uman/evaluare-tehnologii-medicale>

**Serbia:** 1. Positive Lists in Serbia (<https://rfzo.rs/index.php/osiguranalica/lekovi-info/lekovi-actual>); 2. Regulation on the list of medicinal products prescribed and dispensed at the expense of mandatory health insurance funds. Official Gazette of the Republic of Serbia," No. 40/2022, 144/2022, 40/2023, 57/2023, 66/2023,67/2023, 86/2023, 104/2023, 17/2024, 74/2024, and 98/2024 (https://www.paragraf.rs/propisi/pravilnik_o_listi_lekova_koji_se_propisuju_i_izdaju_na_teret_sredstava_obaveznog_zdravstvenog_3. Regulation on the conditions, criteria, method, and procedure for the inclusion of a medicinal product on the list of medicines, amendments and supplements to the list of medicines, or the removal of a medicinal product from the list of medicines. Official Gazette of the Republic of Serbia," No. 41/2014, 125/2014, 48/2015, and 14/2018 (<http://demo.paragraf.rs/demo/combined/Old/t/t2018_02/t02_0347.htm>); 4. SmPC of ibrutinib (<https://www.alims.gov.rs/doc_file/lekovi/smpc/515-01-02462-20-001.pdf>)

**Slovakia:** 1. Slovak MOH website: <https://health.gov.sk/Titulka>

**Ukraine:** 1. <https://www.dec.gov.ua/>; 2. <https://www.dec.gov.ua/wp-content/uploads/2022/02/vysnovok-z-derzhavnoyi-omt-venetoklaks-venkliksto-vid-07.08.2024.pdf>; 3. <https://www.dec.gov.ua/wp-content/uploads/2022/02/vysnovok-z-derzhavnoyi-omt-ibrutynib-vid-26.12.2023-1.pdf>; 4. <https://www.dec.gov.ua/wp-content/uploads/2021/11/venkliksto_vysnovok.pdf>

# Supplementary Tables

**Supplementary Table 1. Selected demographic, macroeconomic, and epidemiological data across the analyzed countries.**

| **Country** | **Demographic** | | **Macroeconomic data** | | | | **CLL epidemiology** | | | |
| --- | --- | --- | --- | --- | --- | --- | --- | --- | --- | --- |
|  | **Population, number^a^** | **People 65+, %^a^** | **Life expectancy, years^b^** | **GDP per capita, US dollars^a^** | **Current health expenditures, % of GDP^b^** | **GGHE-D, % of GGE^b^** | **Prevalence, ratio per 100,000^c^** | **Incidence, ratio per 100,000^c^** | **Deaths, ratio per 100,000^c^** | **DALY, ratio per 100,00^c^** |
| Armenia | 2,962,300 | 12.60% | 73.0 | 4,685.18 | 12,34% | 7.60% | 10.55 | 1.78 | 0.81 | 20.93 |
| Bosnia and Herzegovina | 3,244,907 | 20.68% | 74.8 | 7,295.34 | 9,56% | 16.40% | 24.47 | 4.24 | 1.87 | 40.31 |
| Bulgaria | 6,877,743 | 21.70% | 71.3 | 12,274.08 | 8,56% | 12.90% | 13.80 | 2.70 | 1.40 | 28.61 |
| Croatia | 3,878,981 | 22.10% | 76.9 | 17,789.93 | 8,10% | 14.00% | 64.15 | 10.20 | 3.42 | 65.45 |
| Czech Republic | 10,505,772 | 20.54% | 77.1 | 27,696.46 | 9,49% | 17.60% | 51.78 | 8.10 | 2.69 | 53.13 |
| Estonia | 1,330,932 | 20.43% | 77.1 | 27,953.77 | 7,49% | 13.80% | 61.54 | 9.93 | 3.70 | 74.10 |
| Hungary | 9,709,891 | 20.41% | 74.4 | 18,755.10 | 7,38% | 11.00% | 44.21 | 7.24 | 2.72 | 56.36 |
| Israel | 9,371,400 | 12.20% | 81.7 | 52,255.62 | 7,90% | 13.20% | 34.68 | 5.72 | 2.24 | 37.84 |
| Lithuania | 2,800,839 | 19.95% | 73.9 | 23,934.73 | 7,82% | 14.00% | 40.47 | 7.18 | 3.34 | 67.85 |
| Montenegro | 619,211 | 16.91% | 74.7 | 9,465.96 | 10,55% | 14.40% | 32.79 | 5.24 | 1.83 | 41.04 |
| Poland | 36,981,559 | 18.53% | 75.4 | 18,635.51 | 6,44% | 10.50% | 41.49 | 7.02 | 2.93 | 58.23 |
| Romania | 19,122,059 | 19.33% | 72.8 | 14,986.79 | 6,48% | 12.30% | 20.02 | 3.51 | 1.57 | 32.79 |
| Serbia | 6,834,326 | 21.89% | 72.8 | 9,680.53 | 10,10% | 13.40% | 31.75 | 5.35 | 2.13 | 44.85 |
| Slovakia | 5,447,247 | 17.16% | 74.5 | 22,138.19 | 7,75% | 13.60% | 39.99 | 6.44 | 2.43 | 53.53 |
| Ukraine | 44,298,640 | 17.39% | 70.9 | 4,775.95 | 8,01% | 10.20% | 18.28 | 3.13 | 1.41 | 35.36 |

DALY, disability-adjusted life years; GDP, gross domestic product; GGE, general government expenditure, GGHE-D, domestic general government health expenditure; US, United States
^a^ World Bank Open Data (2021) (World Bank Group, 2021).
^b^ World Health Organization (2021) (World Health Organization, 2021).
^c^ Institute for Health Metrics and Evaluation (2021) (Institute for Health Metrics and Evaluation (IHME), 2022).

**Supplementary Table 2. Marketing authorization and reimbursement indications in R/R CLL across the analyzed countries**

| **Country / organisation** | **Detailed indications** | | | | | | | |
| --- | --- | --- | --- | --- | --- | --- | --- | --- |
|  | **Acalabrutinib** | **Ibrutinib** | | **Zanubrutinib** | **Duvelisib** | **Idelalisib** | **Venetoclax** | |
|  |  | **Monotherapy** | **Combination therapy** |  |  |  | **Monotherapy** | **Combination therapy** |
| **Marketing authorization** | | | | | | | | |
| EMA | Acalabrutinib as monotherapy is indicated for the treatment of adult patients with CLL who have received at least one prior therapy. | Ibrutinib as a single agent is indicated for the treatment of adult patients with CLL who have received at least one prior therapy. | Ibrutinib as a combination with bendamustine and rituximab (BR) is indicated for the treatment of adult patients with CLL who have received at least one prior therapy. | Zanubrutinib as monotherapy is indicated for the treatment of adult patients with CLL. | Duvelisib as monotherapy is indicated for the treatment of adult patients with R/R CLL after at least two prior therapies. | Idelalisib as combination with rituximab for the treatment of adult patients with CLL who have received at least one prior therapy | As monotherapy is indicated for the treatment of CLL in the presence of 17p deletion or TP53 mutation in adult patients who are unsuitable for or have failed a B-cell receptor pathway inhibitor, or in the absence of 17p deletion or TP53 mutation in adult patients who have failed both chemoimmunotherapy and a B-cell receptor pathway inhibitor. | Venetoclax in combination with rituximab is indicated for the treatment of adult patients with CLL who have received at least one prior therapy. |
| **Reimbursement indication** | | | | | | | | |
| Armenia | Not reimbursed | Not reimbursed | Not reimbursed | Not reimbursed | Not reimbursed | Not reimbursed | Not reimbursed | Not reimbursed |
| Bosnia and Herzegovina | Not reimbursed | Ibrutinib as monotherapy is reimbursed in adult patients with CLL who have received at least one previous therapy  **Additional limitations:**  ECOG performance status 0–1, or 0–2 for disabled patients with associated comorbidities, following careful consideration of the risk-benefit ratio. Patients must have favorable prognostic characteristics, including a low tumor burden or less aggressive disease, defined as one or two metastatic sites and a time from diagnosis to first metastasis of ≥18 months. | Not reimbursed | Not reimbursed | Not reimbursed | Not reimbursed | Venetoclax as monotherapy or in combination with rituximab is reimbursed for adult patients with CLL who have received at least one previous therapy. | |
| Bulgaria | Acalabrutinib in monotherapy is reimbursed for adult patients with CLL who have received at least one prior therapy | Ibrutinib, either as monotherapy or in combination with bendamustine and rituximab, is reimbursed for adult patients with CLL who have received at least one previous therapy | | Zanubrutinib is reimbursed for adult patients with CLL | Not reimbursed | Not reimbursed | Venetoclax as monotherapy or in combination with rituximab is reimbursed for adult patients with CLL who have received at least one previous therapy, in accordance with SmPC indications | |
| Croatia | Acalabrutinib in monotherapy is reimbursed for patients **with del17p/mTP53** or for previously treated patients who are **refractory to therapy or are experience early relapse**, defined as a relapse within 24 months from the completion of prior therapy.  **Additional limitations:**  Before initiating therapy, cytogenetic testing, FISH from bone marrow or peripheral blood, and radiological assessment of lymph nodes, liver, and spleen infiltration are mandatory. Patients presenting with symptoms of the disease must meet at least one of the following criteria to qualify for treatment:  a) high-risk status, classified as RAI stage III–IV; b) tumor tissue mass ≥15; c) extensive tumor burden, defined as a single nodule or conglomerate >10 cm, or progressive / symptomatic lymphadenopathy; d) significant B symptoms that impact quality of life, including: unintentional weight loss ≥10% within the last six months, persistent fever >38°C for at least two weeks without evidence of infection, night sweats lasting more than one month without an infectious cause. Additionally, patients must have an ECOG performance status of 0–2. | Ibrutinib in monotherapy is reimbursed for patients **with del17p/mTP53** or for previously treated patients who are **refractory to therapy or are experience early relapse**, defined as a relapse within 24 months from the completion of prior therapy.  **Additional limitations:**  Before initiating therapy, cytogenetic testing, FISH from bone marrow or peripheral blood, and radiological assessment of lymph nodes, liver, and spleen infiltration are mandatory. Patients presenting with symptoms of the disease must meet at least one of the following criteria to qualify for treatment:  a) high-risk status, classified as RAI stage III–IV; b) tumor tissue mass ≥15; c) extensive tumor burden, defined as a single nodule or conglomerate >10 cm, or progressive / symptomatic lymphadenopathy; d) significant B symptoms that impact quality of life, including: unintentional weight loss ≥10% within the last six months, persistent fever >38°C for at least two weeks without evidence of infection, night sweats lasting more than one month without an infectious cause. | Not reimbursed | Not reimbursed | Not reimbursed | Not reimbursed | Venetoclax monotherapy is reimbursed for the treatment of CLL in patients with del17p/mTP53 who are unsuitable for or have not responded to BCR signaling pathway inhibitor, or for patients who experience **early relapse or are refractor**y to both chemoimmunotherapy and an BCR signaling pathway inhibitor.  **Additional limitations:**  Before initiating therapy, cytogenetic testing, FISH from bone marrow or peripheral blood, and radiological assessment of lymph nodes, liver, and spleen infiltration are mandatory. Patients presenting with symptoms of the disease must meet at least one of the following criteria to qualify for treatment:  a) high-risk status, classified as RAI stage III–IV; b) tumor tissue mass ≥15; c) extensive tumor burden, defined as a single nodule or conglomerate >10 cm, or progressive / symptomatic lymphadenopathy; d) significant B symptoms that impact quality of life, including: unintentional weight loss ≥10% within the last six months, persistent fever >38°C for at least two weeks without evidence of infection, night sweats lasting more than one month without an infectious cause. | Venetoclax is reimbursed for the treatment of adult patients with CLL who have received at least one prior therapy and are either **refractory to therapy or in early relapse**, defined as relapse occurring within 24 months of completing the previous therapy  **Additional limitations:**  Before initiating therapy, cytogenetic testing, FISH from bone marrow or peripheral blood, and radiological assessment of lymph nodes, liver, and spleen infiltration are mandatory. Patients presenting with symptoms of the disease must meet at least one of the following criteria to qualify for treatment:  a) high-risk status, classified as RAI stage III–IV; b) tumor tissue mass ≥15; c) extensive tumor burden, defined as a single nodule or conglomerate >10 cm, or progressive / symptomatic lymphadenopathy; d) significant B symptoms that impact quality of life, including: unintentional weight loss ≥10% within the last six months, persistent fever >38°C for at least two weeks without evidence of infection, night sweats lasting more than one month without an infectious cause. |
| Czech Republic | Acalabrutinib monotherapy is reimbursed for adult patients with CLL and an ECOG PS of 0–1 who meet at least one of the following criteria:   - are **refractory** to recent therapy; - they **have relapsed within 24 months** of the previous treatment; - they have relapsed and are **unsuitable for chemoimmuno-therapy**; - have a presence of **del17p/mTP53**   **Additional limitations:**  Reimbursement limited to centers of excellence | Ibrutinib monotherapy is reimbursed for adult patients with CLL and an ECOG PS of 0–1 who meet at least one of the following criteria:   - are **refractory** to recent therapy; - they **have relapsed within 24 months** of the previous treatment; - they have relapsed and are **unsuitable for chemoimmuno-therapy**; - have a presence of **del17p/mTP53**   **Additional limitations:**  Reimbursement limited to centers of excellence | Not reimbursed | Zanubrutinib monotherapy is reimbursed for adult patients with CLL and an ECOG PS of 0–1 who meet at least one of the following criteria:   - are **refractory** to recent therapy; - they **have relapsed within 24 months** of the previous treatment; - they have relapsed and are **unsuitable for chemoimmuno-therapy**; - have a presence of **del17p/mTP53**   **Additional limitations:**  Reimbursement limited to centers of excellence | Not reimbursed | Idelalisib, in combination with rituximab, is reimbursed for the treatment of adult patients with R/R CLL who have ECOG PS 0–2 and have received at least one prior line of chemo(immuno)-therapy, and experience **early disease progression or early relapse** (within 24 months of completion of prior therapy) **and for whom further treatment with cytotoxic chemo(immuno)-therapy is not considered appropriate** **due to adverse disease cytogenetics** (del17p/mTP53) **or general health** (comorbidities, advanced age)  **Additional limitations:**  Reimbursement limited to centers of excellence | Not reimbursed | Venetoclax is reimbursed in combination with rituximab is reimbursed for adult patients with CLL and an ECOG PS of 0–1 who meet at least one of the following criteria:   - are **refractory** to recent therapy; - they **have relapsed within 24 months** of the previous treatment; - they have relapsed and are **un suitable for chemoimmuno-therapy**; - have a presence of **del17p/mTP53**   **Additional limitations:**  Reimbursement limited to centers of excellence |
| Estonia | Acalabrutinib monotherapy is reimbursed for patients with CLL who have **del17p/mTP53 mutation** and have received **only one prior therapy, with the disease being refractory** or **relapsing within 36 months** of the last treatment, or for those **with del11q who have received only one prior therapy** and experienced refractory disease or relapse | Ibrutinib monotherapy is reimbursed for patients with CLL who have **del17p/mTP53 mutation** and have received **only one prior therapy, with the disease being refractory** or **relapsing within 36 months** of the last treatment, or for those **with del11q who have received only one prior therapy** and experienced refractory disease or relapse | Not reimbursed | Not reimbursed | Not reimbursed | Not reimbursed | Not reimbursed | Venetoclax in combination with rituximab is reimbursed for patients with CLL who have received at least one prior therapy |
| Hungary | Acalabrutinib is reimbursed only for patients:   - - without del17p/mTP53 who experience relapse **after one cycle of treatment**;   - with **at least two relapses or progressions, occurring no later than 12 months** after the last relapse or progression;   - with **del17p/mTP53 or unmutaed IgVH if they are ineligible for allogenic stem cell transplant** | Ibrutinib is reimbursed only for patients:   - - without del17p/mTP53 who experience relapse **after one cycle of treatment**;   - with **at least two relapses or progressions, occurring no later than 12 months** after the last relapse or progression;   - with **del17p/mTP53 or unmutaed IgVH if they are ineligible for allogenic stem cell transplant** | Not reimbursed | Not reimbursed | Not reimbursed | Not reimbursed | Venetoclax as monotherapy or in combination with rituximab is reimbursed for adult patients with CLL who have received at least one previous therapy, in accordance with SmPC indications | |
| Israel | Acalabrutinib, as monotherapy or in combination with obinutuzumab, is reimbursed for the treatment of CLL in patients whose disease relapsed or was refractory to prior treatment with a regimen including bendamustine in combination with rituximab, FCR, obinutuzumab, chlorambucil with an anti-CD20 antibody, or venetoclax.  **Additional limitations:**  Patients are entitled to treatment with only one BTKi during the disease course, except for those who received a time-limited first-line combination of ibrutinib and venetoclax, who remain eligible for an additional line of BTKi monotherapy | Ibrutinib monotherapy is reimbursed for the treatment of CLL in patients whose disease relapsed or was refractory to prior treatment with a regimen including bendamustine in combination with rituximab, FCR, obinutuzumab, chlorambucil with an anti-CD20 antibody, or venetoclax.  **Additional limitations:**  Patients are entitled to treatment with only one BTKi during the disease course, except for those who received a time-limited first-line combination of ibrutinib and venetoclax, who remain eligible for an additional line of BTKi monotherapy | Not reimbursed | Not reimbursed | Not reimbursed | Not reimbursed | Venetoclax, as monotherapy or in combination with rituximab, is reimbursed for the treatment of CLL in patients whose disease has relapsed or was refractory to prior treatment.  **Additional limitations:**  The treatment is reimbursed only for patients who have not previously been treated with venetoclax for their disease. | |
| Lithuania | Acalabrutinib monotherapy is reimbursed for patients with R/R CLL who have received at least one prior treatment, provided that the previous regimen is not repeated and **relapse occurs within 36 months of completing first-line therapy**. | Ibrutinib monotherapy is reimbursed for patients with R/R CLL who have received at least one prior treatment, provided that the previous regimen is not repeated and **relapse occurs within 36 months of completing first-line therapy**. | Not reimbursed | Zanubrutinib monotherapy is reimbursed for patients with R/R CLL who have received at least one prior treatment, provided that the previous regimen is not repeated and **relapse occurs within 36 months of completing first-line therapy**. | Not reimbursed | Not reimbursed | Venetoclax monotherapy is reimbursed for the treatment of CLL in adult patients with del17p/mTP53 who are unsuitable for or have failed a BCR signaling pathway inhibitor, or in those without del17p/mTP53 who have failed both chemoimmunotherapy and a BCR signaling pathway inhibitor | Venetoclax, in combination with rituximab, is reimbursed for the treatment of CLL in patients who have previously received at least one line of CLL therapy, provided that disease relapse occurs within **36 months after first-line treatment**, without repeating the previous regimen. |
| Montenegro | Not reimbursed | Ibrutinib, as monotherapy or in combination with bendamustine and rituximab, is reimbursed for adult patients with CLL who have previously received at least one therapy. | | Not reimbursed | Not reimbursed | Not reimbursed | Not reimbursed | Not reimbursed |
| Poland | Acalabrutinib in monotherapy is reimbursed for the treatment of relapsed or refractory CLL in patients who have previously received at least one line of therapy, regardless of del17p/mTP53 status, **provided they meet the eligibility criteria of the drug program** (age 18 years or older; ECOG performance status 0-2; diagnosis of CLL; presence of indications for treatment according to the International Workshop on Chronic Lymphocytic Leukemia updating (the National Cancer Institute-Working Group; no contraindications to the use of the drug in accordance with the current SmPC; no hypersensitivity to any drug, mouse proteins, or any excipients of the medication; exclusion of pregnancy and breastfeeding; patient's agreement to use contraception in accordance with the appropriate, current SmPC; absence of active, severe infections; absence of significant comorbidities or clinical conditions constituting a contraindication to therapy as determined by the treating physician based on the appropriate, current SmPC; adequate organ function as determined by blood laboratory test results, allowing for the safe initiation of therapy in the opinion of the treating physician) | Ibrutinib in monotherapy is indicated for the treatment of relapsed or refractory CLL in patients who have previously received at least one line of therapy, regardless of del17p/mTP53 status, **provided they meet the eligibility criteria of the drug program** (age 18 years or older; ECOG performance status 0-2; diagnosis of CLL; presence of indications for treatment according to the International Workshop on Chronic Lymphocytic Leukemia updating (the National Cancer Institute-Working Group; no contraindications to the use of the drug in accordance with the current SmPC; no hypersensitivity to any drug, mouse proteins, or any excipients of the medication; exclusion of pregnancy and breastfeeding; patient's agreement to use contraception in accordance with the appropriate, current SmPC; absence of active, severe infections; absence of significant comorbidities or clinical conditions constituting a contraindication to therapy as determined by the treating physician based on the appropriate, current SmPC; adequate organ function as determined by blood laboratory test results, allowing for the safe initiation of therapy in the opinion of the treating physician) | Not reimbursed | Zanubrutinib in monotherapy is indicated for the treatment of relapsed or refractory CLL in patients who have previously received at least one line of therapy, regardless of del17p/mTP53 status, **provided they meet the eligibility criteria of the drug program** (age 18 years or older; ECOG performance status 0-2; diagnosis of CLL; presence of indications for treatment according to the International Workshop on Chronic Lymphocytic Leukemia updating (the National Cancer Institute-Working Group; no contraindications to the use of the drug in accordance with the current SmPC; no hypersensitivity to any drug, mouse proteins, or any excipients of the medication; exclusion of pregnancy and breastfeeding; patient's agreement to use contraception in accordance with the appropriate, current SmPC; absence of active, severe infections; absence of significant comorbidities or clinical conditions constituting a contraindication to therapy as determined by the treating physician based on the appropriate, current SmPC; adequate organ function as determined by blood laboratory test results, allowing for the safe initiation of therapy in the opinion of the treating physician) | Not reimbursed | Not reimbursed | Venetoclax in monotherapy is reimbursed in patients with CLL and **del17p/mTP53 who** **have failed BTKi treatment**, **provided they meet the eligibility criteria of the drug program** (age 18 years or older; ECOG performance status 0-2; diagnosis of CLL; presence of indications for treatment according to the International Workshop on Chronic Lymphocytic Leukemia updating (the National Cancer Institute-Working Group; no contraindications to the use of the drug in accordance with the current SmPC; no hypersensitivity to any drug, mouse proteins, or any excipients of the medication; exclusion of pregnancy and breastfeeding; patient's agreement to use contraception in accordance with the appropriate, current SmPC; absence of active, severe infections; absence of significant comorbidities or clinical conditions constituting a contraindication to therapy as determined by the treating physician based on the appropriate, current SmPC; adequate organ function as determined by blood laboratory test results, allowing for the safe initiation of therapy in the opinion of the treating physician) | Venetoclax in combination with rituximab is reimbursed for the treatment of relapsed or refractory CLL in patients who have previously received at least one line of therapy, regardless of del17p/mTP53 status, **provided they meet the eligibility criteria of the drug program** (age 18 years or older; ECOG performance status 0-2; diagnosis of CLL; presence of indications for treatment according to the International Workshop on Chronic Lymphocytic Leukemia updating (the National Cancer Institute-Working Group; no contraindications to the use of the drug in accordance with the current SmPC; no hypersensitivity to any drug, mouse proteins, or any excipients of the medication; exclusion of pregnancy and breastfeeding; patient's agreement to use contraception in accordance with the appropriate, current SmPC; absence of active, severe infections; absence of significant comorbidities or clinical conditions constituting a contraindication to therapy as determined by the treating physician based on the appropriate, current SmPC; adequate organ function as determined by blood laboratory test results, allowing for the safe initiation of therapy in the opinion of the treating physician) |
| Romania | Acalabrutinib in monotherapy is reimbursed for the treatment of adult patients with CLL who have received at least one prior line of therapy | Ibrutinib as monotherapy or in combination with bendamustine and rituximab, is reimbursed for patients who have received at least one prior treatment. | | Not reimbursed | Not reimbursed | Not reimbursed | Venetoclax monotherapy is reimbursed for the treatment of CLL in adult patients with del17p/mTP53 who are unsuitable for or have failed a BCR signaling pathway inhibitor, or in those without del17p/mTP53 who have failed both chemoimmunotherapy and a BCR signaling pathway inhibitor | Not reimbursed |
| Serbia | Not reimbursed | Ibrutinib, as monotherapy or in combination with bendamustine and rituximab, is reimbursed for patients with R/R CLL who have:   - have del17p or mTP53 or - not responded to prior therapy or have experienced **early relapse** (within 36 months from the start of therapy) or; - received **at least two lines of therapy** with good overall functional status (ECOG PS 0–1). | | Not reimbursed | Not reimbursed | Not reimbursed | Not reimbursed | Not reimbursed |
| Slovakia | Not reimbursed | Ibrutinib in monotherapy is reimbursed for adult patients with R/R CLL with an ECOG PS 0–2, an absolute neutrophil count ≥0,75 x 10^9^and a platelet count ≥30 x 10^9^, who either:   - are **refractory or have relapsed within 18 month** of previous therapy, or - **have a presence of del17p/mTP53**   **Additional limitations:**  Ibrutinib can only be prescribed by designated healthcare providers. | Not reimbursed | Not reimbursed | Not reimbursed | Not reimbursed | Venetoclax monotherapy is reimbursed for the treatment of CLL in adult patients with del17p/mTP53 who are unsuitable for or have failed a BCR signaling pathway inhibitor, or in those without del17p/mTP53 who have failed both chemoimmunotherapy and a BCR signaling pathway inhibitor  **Additional limitations:**  Venetoclax can only be prescribed by designated healthcare providers. | Venetoclax in combination with rituximab is reimbursed for adult patients with R/R CLL with an ECOG PS 0–2, an absolute neutrophil count ≥0,75 x 10^9^and a platelet count ≥30 x 10^9^, who either:   - are **refractory or have relapsed within 18 month** of previous therapy, or   **have a presence of del17p/mTP53**  **Additional limitations:**  Venetoclax can only be prescribed by designated healthcare providers. |
| Ukraine | Not reimbursed | Not reimbursed | Not reimbursed | Not reimbursed | Not reimbursed | Not reimbursed | Not reimbursed | Not reimbursed |

Blue cells indicate full alignment with the SmPC; orange cells represent restrictions in the reimbursement indication.
BCR, B-cell receptor; BTKi, Bruton’s tyrosine kinase inhibitor; CLL, chronic lymphocytic leukemia; del17p, deletion 17p; ECOG, Eastern Cooperative Oncology Group; EMA, European Medicines Agency; mTP53, TP53 gene mutation; PS, performance status; R/R, relapsed/refractory; SmPC, summary of product characteristics

**Supplementary Table 3. Official prices and costs of targeted therapies for R/R CLL across the analyzed countries.**

| **Country** | **Package** | **Price type** | **Current prices** | | **Therapy costs** | | | | **Price changes** | |
| --- | --- | --- | --- | --- | --- | --- | --- | --- | --- | --- |
|  |  |  | **National currency** | **Euro** | **Per capsule/tablet** | **Daily** | **Monthly (30 days)** | **Yearly (365 days)** | **Since the initial decision** | **%** |
| Acalabrutinib (Calquence^®^) 100 mg, daily dose 2 x 100 mg | | | | | | | | | | |
| Bulgaria | 56 tablets or hard capsules | Manufacturer | 11,073.40 BGN | 5,661.83 EUR | 101.10 EUR | 202.21 EUR | 6,066.24 EUR | 73,805.95 EUR | Stable | N/A |
|  |  | Wholesale | 13,300.08 BGN | 6,800.33 EUR | 121.43 EUR | 242.87 EUR | 7,286.06 EUR | 88,647.12 EUR |  | N/A |
|  |  | Pharmacy price | 13,330.08 BGN | 6,815.67 EUR | 121.71 EUR | 243.42 EUR | 7,302.50 EUR | 88,847.08 EUR |  | N/A |
| Croatia | 56 tablets or hard capsules | Official price | 4,765.37 EUR | 4,765.37 EUR | 85.10 EUR | 170.19 EUR | 5,105.75 EUR | 62,120.00 EUR | Stable | N/A |
| Czech Republic | 56 tablets or hard capsules | Official price (incl. margins and VAT) | 112,608.63 CZK | 4,482.83 EUR | 80.05 EUR | 160.10 EUR | 4803.03 EUR | 58,436.86 EUR | Decrease | -12% |
| Estonia | 60 hard capsules | Official price (retail) | 5,463.00 EUR | 5,463.00 EUR | 91.05 EUR | 182.10 EUR | 5,463.00 EUR | 66,466.50 EUR | Stable | N/A |
| Hungary | 60 hard capsules | List manufacturer price | 2,012,099.00 HUF | 5,090.06 EUR | 84.83 EUR | 169.67 EUR | 5,090.06 EUR | 61,929.01 EUR | Stable | N/A |
| Israel | 60 hard capsules | Official price | 25,806.60 NIS | 6,440.86 EUR | 107.35 EUR | 214.70 EUR | 6,440.86 EUR | 78,363.82 EUR | Decrease | -11% |
| Lithuania | 60 hard capsules | Reimbursement price | 3.148.83 EUR | 3.148.83 EUR | 52.48 EUR | 104.96 EUR | 3,148.83 EUR | 38,310.76 EUR | Stable | N/A |
| Poland | 56 tablets or hard capsules | Net selling price | 23,000.00 PLN | 5,341,63 EUR | 95.39 EUR | 190.77 EUR | 5,723.18 EUR | 69,631.99 EUR | Stable | N/A |
|  |  | Official price | 24,840.00 PLN | 5,768,96 EUR | 103.02 EUR | 206.03 EUR | 6,181.03 EUR | 75,202.55 EUR |  |  |
|  |  | Wholesale | 26,330.40 PLN | 6,115.10 EUR | 109.20 EUR | 218.40 EUR | 6,551.89 EUR | 79,714.70 EUR |  |  |
| Romania | 60 hard capsules | Manufacturer (ex-factory) | 24,704.23 RON | 4,966.07 EUR | 82.77 EUR | 165.54 EUR | 4,966.07 EUR | 6,0420.56 EUR | Increase | +20% |
|  |  | Retail (includes WHS, pharmacy margins, VAT 9%) | 27,540.03 RON | 5,536.13 EUR | 92.27 EUR | 184.54 EUR | 5,536.13 EUR | 6,7356.24 EUR |  |  |
| Ibrutinib (Imbruvica^®^) 140 mg, daily dose 3 x 140 mg | | | | | | | | | | |
| Bosnia and Herzegovina | 90 capsules | Official price | 5,212.80 EUR | 5,212.80 EUR | 57.92 EUR | 173.76 EUR | 5,212.80 EUR | 63,422.40 EUR | Decrease | -7% |
| Bulgaria | 90 capsules | Manufacturer | 9,343.20 BGN | 4,777.18 EUR | 53.08 EUR | 159.24 EUR | 4,777.18 EUR | 58,122.30 EUR | Decrease | -10% |
|  |  | Wholesale | 11,223.84 BGN | 5,738.75 EUR | 63.76 EUR | 191.29 EUR | 5,738.75 EUR | 69,821.41 EUR |  |  |
|  |  | Pharmacy | 11,253.84 BGN | 5,754.09 EUR | 63.93 EUR | 191.80 EUR | 5,754.09 EUR | 70,008.04 EUR |  |  |
| Croatia | 90 capsules | Official price | 5,377.00 EUR | 5,377.00 EUR | 59.74 EUR | 179.23 EUR | 5,377.00 EUR | 65,420.17 EUR | Stable | N/A |
| Czech Republic | 90 capsules | Official price (incl. margins and VAT) | 120,652.10 CZK | 4,803.03 EUR | 53.37 EUR | 160.10 EUR | 4,803.03 EUR | 58,436.86 EUR | Decrease | N/A |
| Estonia | 30 capsules | Official price | 2,010.86 EUR | 2,010.86 EUR | 67.03 EUR | 201.09 EUR | 6,032.58 EUR | 73,396.39 EUR | Stable | N/A |
| Israel | 90 capsules | Official price | 24,490.00 NIS | 6,112.26 EUR | 67.91 EUR | 203.74 EUR | 6,112.26 EUR | 74,365.85 EUR | Decrease | -15% |
| Hungary | 28 tablets | List manufacturer price | 514,857.00 HUF | 1,302.45 EUR | 46.52 EUR | 139.55 EUR | 4,186.43 EUR | 50,934.95 EUR | Stable | N/A |
| Lithuania | 30 tablets | Reimbursement price | 1,596.72 EUR | 1,562.48 EUR | 52.08 EUR | 156.25 EUR | 4,687.44 EUR | 57,030,52 EUR | Decrease | -6% |
| Montenegro | 90 capsules | Maximum wholesale | 4,907.26 EUR | 4,907.26 EUR | 54.53 EUR | 163.58 EUR | 4,907.26 EUR | 59,705.00 EUR | Decrease | -12% |
| Poland | 90 capsules | Net selling price | 21,600.00 PLN | 5,016.49 EUR | 55.74 EUR | 167.22 EUR | 5,016.49 EUR | 61,033.95 EUR | Decrease | -6% |
|  |  | Official price | 23,328.00 PLN | 5,417.81 EUR | 60.20 EUR | 180.59 EUR | 5,417.81 EUR | 65,916.67 EUR |  |  |
|  |  | Wholesale | 24,727.68 PLN | 5,742,88 EUR | 63.81 EUR | 191.43 EUR | 5,742.88 EUR | 69,871.67 EUR |  |  |
| Romania | 30 capsules | Retail (includes WHS, pharmacy margins, VAT 9%) | 7,658.64 RON | 1,539.55 EUR | 51.32 EUR | 153.95 EUR | 4,618.65 EUR | 56,193.54 EUR | Decrease | -5% |
|  |  | Manufacturer (ex-factory) | 8,586.89 RON | 1,726.15 EUR | 57.54 EUR | 172.61 EUR | 5,178.44 EUR | 63,004.36 EUR |  |  |
| Serbia | 90 capsules | Wholesale | 594,087.80 RSD | 5,073.34 EUR | 56.37 EUR | 169.11 EUR | 5,073.34 EUR | 61,725.60 EUR | Stable | N/A |
| Slovakia | 30 capsules | Maximal/reimbursement price | 1,901.90 EUR | 1,901.90 EUR | 63.40 EUR | 190,19 EUR | 5,705.70 EUR | 69,419.35 EUR | Stable | N/A |
| Zanubrutinib (Brukinsa^®^) 80 mg, daily dose 4 x 80 mg | | | | | | | | | | |
| Bulgaria | 120 hard capsules | Manufacturer | 9,661.72 BGN | 4,940.03 EUR | 41.17 EUR | 164.67 EUR | 4,940.03 EUR | 60,103.76 EUR | Stable | N/A |
|  |  | Wholesaler | 11,606.06 BGN | 5,934.18 EUR | 49.45 EUR | 197.81 EUR | 5,934.18 EUR | 72,199.13 EUR |  |  |
|  |  | Pharmacy | 11,636.06 BGN | 5,9349.51 EUR | 49.58 EUR | 198.32 EUR | 5,949.51 EUR | 72,385.76 EUR |  |  |
| Czech Republic | 120 hard capsules | Official price (incl. margins and VAT) | 116,663.03 CZK | 4,644.23 EUR | 38.70 EUR | 154.81 EUR | 4,644.23 EUR | 56,504.78 EUR | Stable | N/A |
| Lithuania | 120 hard capsules | Reimbursement price | 3,074.52 EUR | 3,074.52 EUR | 25.62 EUR | 102.48 EUR | 3,074.52 EUR | 37 406,66 EUR | Decrease | -3% |
| Poland | 120 hard capsules | Net selling price | 21,552.00 PLN | 5,005.34 EUR | 41.71 EUR | 166.84 EUR | 5,005.34 EUR | 60,898.32 EUR | Stable | N/A |
|  |  | Official price | 23,276.16 PLN | 5,405.77 EUR | 45.05 EUR | 180.19 EUR | 5,405.77 EUR | 65,770.19 EUR |  |  |
|  |  | Wholesale | 24,672.73 PLN | 5,730.12 EUR | 47.75 EUR | 191.00 EUR | 5,730.12 EUR | 69,716.40 EUR |  |  |
| Venetoclax (Venclyxto^®^) 100 mg, daily dose 4 x 100 mg | | | | | | | | | | |
| Bosnia and Herzegovina | 112 tablets | Official price | 5,150.88 EUR | 5,150.88 EUR | 45.99 EUR | 183.96 EUR | 5,518.80 EUR | 67,145.40 EUR | Stable | N/A |
| Bulgaria | 112 tablets | Manufacturer | 8,596.58 BGN | 4,395.43 EUR | 39.24 EUR | 156.98 EUR | 4,709.39 EUR | 57,297.56 EUR | Decrease | -25% |
|  |  | Wholesale | 10,327.90 BGN | 5,280.65 EUR | 47.15 EUR | 188.59 EUR | 5,657.84 EUR | 68,837.08 EUR |  |  |
|  |  | Pharmacy | 10,357.90 BGN | 5,295.99 EUR | 47.29 EUR | 189.14 EUR | 5,674.28 EUR | 69,037.03 EUR |  |  |
| Croatia | 112 tablets | Official price | 5.660,00 EUR | 5.660,00 EUR | 50.54 EUR | 202.14 EUR | 6,064.29 EUR | 7,3782.14 EUR | Stable | N/A |
| Czech Republic | 112 tablets | Official price (incl. margins and VAT) | 134,132.07 CZK | 5,339.65 EUR | 47.68 EUR | 190.70 EUR | 5,721.06 EUR | 69,606.18 EUR | Decrease | N/A |
| Estonia | 112 tablets | Official price | 6,407.84 EUR | 6,407.84 EUR | 57.21 EUR | 228.85 EUR | 6,865.54 EUR | 83,530.77 EUR | Stable | N/A |
| Hungary | 112 tablets | List manufacturer price | 1,662,548.00 HUF | 4,205.79 EUR | 37.55 EUR | 150.21 EUR | 4,506.20 EUR | 5,4825.45 EUR | Stable | N/A |
| Israel | 120 tablets | Official price | 26,515.78 NIS | 6,617.86 EUR | 55.15 EUR | 220.60 EUR | 6,617.86 EUR | 8,0517.30 EUR | Decrease | -23% |
| Lithuania | 112 tablets | Reimbursement prices | 3,681.84 EUR | 3,681.84 EUR | 32.87 EUR | 131.49 EUR | 3 944,70 EUR | 47,993.85 EUR | Decrease | -32% |
| Poland | 112 tablets | Net selling price | 19,130.40 PLN | 4,442.94 EUR | 39.67 EUR | 158.68 EUR | 4,760.29 EUR | 57,916.86 EUR | Decrease | -23% |
|  |  | Official price | 20,660.83 PLN | 4,798.37 EUR | 42.84 EUR | 171.37 EUR | 5,141.11 EUR | 62,550.21 EUR |  |  |
|  |  | Wholesale | 21,900.48 PLN | 5,086.27 EUR | 45.41 EUR | 181.65 EUR | 5,449.58 EUR | 66,303.22 EUR |  |  |
| Romania | 112 tablets | Retail (includes WHS, pharmacy margins, VAT 9%) | 22,697.41 RON | 4,562.66 EUR | 40.74 EUR | 162.95 EUR | 4,888.56 EUR | 59,477.54 EUR | Decrease | -22% |
| Slovakia | 112 tablets | Maximal/reimbursement price | 5,135.36 EUR | 5,135.36 EUR | 45.85 EUR | 183.41 EUR | 5,502.17 EUR | 66,943.09 EUR | Stable | N/A |
| Idealisib (Zydelig) 150 mg, daily dose 2 x 150 mg | | | | | | | | | | |
| Czech Republic | 60 tablets | Official price (incl. margins and VAT) | 102,345.35 CZK | 4,074.26 EUR | 67.90 EUR | 135.81 EUR | 4,074.26 EUR | 49,570.13 EUR | Decrease | N/A |

The costs in EUR were calculated using the average annual exchange rates for 2023: 1 EUR = 4.0067 ILS; 1 EUR = 1.9558 BGN; 1 EUR = 25.120 CZK; 1 EUR = 395.30 HUF; 1 EUR = 4.9746 RON; 1 EUR = 4.3058 PLN; 1 EUR = 117.10 RSD.

**Supplementary Table 4. Summary of HTA for targeted therapies processes across analzyed countries**

| **Country** | **Drug** | **Comparator** | **Type of comparison** | **Clinical superiority** | **Economic evaluation** | | | **Time from HTA to reimbursement** | **RSS** |
| --- | --- | --- | --- | --- | --- | --- | --- | --- | --- |
|  |  |  |  |  | **Type** | **Cost-effectiveness** | **ICER/ICUR** |  |  |
| Bulgaria | Acalabrutinib | Ibrutinib | No information | No information | CUA, CEA | Demonstrated | Not publicly available | < 6 months | No information |
|  |  | Ibrutinib+BR | No information | No information | CUA, CEA | Demonstrated | Not publicly available |  |  |
|  |  | Venetoclax | No information | No information | CUA, CEA | Demonstrated | Not publicly available |  |  |
|  |  | Venetoclax+rituximab | No information | No information | CUA, CEA | Demonstrated | Not publicly available |  |  |
|  | Ibrutinib | Ofatumumab | Direct | Demonstrated | CUA, CEA | Not demonstrated | Not publicly available | <6 months | No information |
|  |  | Fludarabine | Indirect (naïve) | Demonstrated | CUA, CEA | Not demonstrated | Not publicly available |  |  |
|  |  | Bendamustine | Indirect (naïve) | Demonstrated | CUA, CEA | Not demonstrated | Not publicly available |  |  |
|  |  | BFR | Indirect (naïve) | Demonstrated | CUA, CEA | Not demonstrated | Not publicly available |  |  |
|  | Ibrutinib  +bendamustine  +rituximab | Ofatumumab | Direct | Demonstrated | CUA, CEA | Not demonstrated | Not publicly available | <6 months | No information |
|  |  | Fludarabine | Indirect (naïve) | Demonstrated | CUA, CEA | Not demonstrated | Not publicly available |  |  |
|  |  | Bendamustine | Indirect (naïve) | Demonstrated | CUA, CEA | Not demonstrated | Not publicly available |  |  |
|  |  | BFR | Indirect (naïve) | Demonstrated | CUA, CEA | Not demonstrated | Not publicly available |  |  |
|  | Venetoclax | BR | No information | No information | CUA | Demonstrated | Not publicly available | <6 months | No information |
|  |  | BO | No information | No information | CUA | Demonstrated | Not publicly available |  |  |
|  |  | Ibrutinib | No information | No information | CUA | Demonstrated | Not publicly available |  |  |
|  |  | BSC | Indirect (naïve) | Demonstrated | CUA | Demonstrated | Not publicly available |  |  |
|  | Venetoclax  +rituximab | BR | No information | No information | CUA | Demonstrated | Not publicly available | <6 months | No information |
|  |  | BO | No information | No information | CUA | Demonstrated | Not publicly available |  |  |
|  |  | Ibrutinib | No information | No information | CUA | Demonstrated | Not publicly available |  |  |
|  |  | BSC | Indirect (naïve) | Demonstrated | CUA | Demonstrated | Not publicly available |  |  |
|  | Zanubrutinib | Ibrutinib | Direct | Demonstrated | CEA, CUA | Not demonstrated | Not publicly available | <6 months | No information |
|  |  | Acalabrutinib | No information | No information | CEA, CUA | Not demonstrated | Not publicly available |  |  |
|  |  | Venetoclax+rituximab | No information | No information | CEA, CUA | Not demonstrated | Not publicly available |  |  |
|  |  | BR | No information | No information | CEA, CUA | Not demonstrated | Not publicly available |  |  |
| Czech Republic | Acalabrutinib | Ibrutinib | Indirect (naïve) | Not applicable (HTA did not assess clinical superiority) | CMA | Not applicable | Not applicable | <6 months | Implemented (details not publicly available, simple discount and budget cap) |
|  |  | Venetoclax+rituximab | Indirect (naïve) | Not applicable (HTA did not assess clinical superiority) | CMA | Not applicable | Not applicable |  |  |
|  | Ibrutinib | Temsirolimus | Indirect (MAIC) | Demonstrated | CEA, CUA | Not demonstrated | 1.8M CZK/QALY  (71,656 EUR/QALY) | <6 months | Implemented (details not publicly available, discount in confidence and budget cap) |
|  |  | BSC | Indirect (MAIC) | Demonstrated | CEA, CA | Not demonstrated | 5.5M CZK/QALY  (218,949 EUR/QALY) |  |  |
|  | Idelalisib  +rituximab | Rituximab | Direct | Demonstrated | CUA | Demonstrated | 782 400 CZK/QALY  (31,146 EUR/QALY) | <6 months | Implemented (details not publicly available, small discount and budget cap) |
|  | Venetoclax  +rituximab | FCR | Indirect (MAIC) | Uncertain (not publicly available) | CUA | Demonstrated | 917,353 CZK/QALY  (36,519 EUR/QALY) | <6 months | Implemented (details not publicly available, most probably a discount and budget cap) |
|  |  | RCD | Indirect (MAIC) | Uncertain (not publicly available) | CUA | Demonstrated | 629,251 CZK/QALY  (25,050 EUR/QALY) |  |  |
|  |  | Ibrutinib | Indirect (MAIC) | Uncertain (not publicly available) | CUA | Demonstrated | Dominant |  |  |
|  |  | Idelalisib+rituximab | Indirect (MAIC) | Uncertain (not publicly available) | CUA | Demonstrated | 335,675 CZK/QALY  (13,363 EUR/QALY) |  |  |
| Estonia | Acalabrutinib | Ibrutinib | Direct | Not applicable (HTA did not assess clinical superiority) | CMA | Not applicable | Not applicable | 6 months to 1 year | Not implemented |
|  | Ibrutinib | Chlorambucil | Direct | Not applicable (HTA did not assess clinical superiority) | CEA, CUA | Demonstrated | Not publicly available | 6 months to 1 year | Not implemented |
|  |  | Chlorambucil+rituximab | Direct | Not applicable (HTA did not assess clinical superiority) | CEA, CUA | Demonstrated | Not publicly available |  |  |
|  | Venetoclax +rituximab | Ibrutinib | Indirect | Not applicable (HTA did not assess clinical superiority | CEA, CUA | Demonstrated | Not publicly available | 6 months to 1 year | Not implemented |
| Hungary | Acalabrutinib | Venetoclax+rituximab | Indirect (NMA) | Not demonstrated | Not performed | Not applicable | Not applicable | 6 months to 1 year | Not implemented |
|  |  | Ibrutinib | Indirect (MAIC) | Not demonstrated | CUA | Demonstrated | Not publicly available |  |  |
| Lithuania | Ibrutinib | Chlorambucil | Indirect | Demonstrated | CEA | Demonstrated | 40,600 EUR/QALY | 6 months to 1 year | Yes (not publicly available) |
|  | Venetoclax  +rituximab | BR | Direct | Demonstrated | CEA | Demonstrated | Not publicly available | 6 months to 1 year | Yes (not publicly available) |
|  | Zanubrutinib | TBC | TBC | TBC | TBC | TBC | TBC | TBC | TBC |
| Poland | Acalabrutinib | Ibrutinib | Direct | Not demonstrated | CMA | Not applicable | Not applicable | 1 to 2 years | Yes (not publicly available) |
|  |  | Venetoclax+rituximab | Indirect (NMA, MAIC, Bucher) – based on subpopulation | Not demonstrated | CMA | Not applicable | Not applicable |  |  |
|  | Ibrutinib | BR | Indirect (MAIC, naive) | Demonstrated | CUA | Not demonstrated | 272,469 PLN/QALY (63,280 EUR/QALY) | 1 to 2 years | Yes (not publicly available) |
|  |  | HDMP+rituximab | Indirect (naïve) | Uncertain | Not performed | Not applicable | Not applicable |  |  |
|  |  | Ofatumumab | Direct | Demonstrated | CUA | Not demonstrated | 291,901 PLN/QALY  (67,792 EUR/QALY) |  |  |
|  |  | SOC | Indirect (Bucher) | Demonstrated | CUA | Demonstrated | Not publicly available |  |  |
|  |  | Venetoclax+rituximab | Indirect (naïve, Bucher) | Not demonstrated | CMA | Not applicable | Not applicable |  |  |
|  | Zanubrutinib | Ibrutinib | Direct | Demonstrated | CUA | Not publicly available | Not publicly available | <6 months | No information |
|  |  | Acalabrutinib | Indirect (MAIC) | Uncertain | CUA | Not publicly available | Not publicly available |  |  |
|  |  | Venetoclax | Indirect (naïve) | Uncertain | CCA | Not applicable | Not applicable |  |  |
|  |  | Venetoclax+rituximab | Indirect (MAIC) | Uncertain | CMA | Not applicable | Not applicable |  |  |
|  |  | BR | Indirect (MAIC) | Uncertain | CUA | Not publicly available | Not publicly available |  |  |
|  | Venetoclax | BSC | Indirect (naïve) | Uncertain | CUA | Not demonstrated | After IBR:  207,779 PLN/QALY (48,256 EUR/QALY)  After BCRi:  216,438 PLN/QALY  (50 267 EUR/QALY) | 1 to 2 years | Yes (not publicly available) |
|  | Venetoclax  +rituximab | BR | Direct | Demonstrated | CUA | Not publicly available | Not publicly available | 6 months to 1 year | No information |
|  |  | Ibrutinib | Indirect (NMA, MAIC, naive) | Uncertain | CUA | Not publicly available | Not publicly available |  |  |
|  | Idelalisib  +rituximab | BR | Indirect (naïve) | Uncertain | CUA | Not demonstrated | 634,119 PLN/QALY  (147,271 EUR/QALY) | Not reimbursed | Implemented (details not publicly available) |
|  |  | Chlormabucil+rituximab | Indirect (naïve) | Uncertain | CUA | Not demonstrated | 715,024 PLN/QALY  (166,061 EUR/QALY) |  |  |
|  |  | HDMP+rituximab | Indirect (naïve) | Uncertain | CUA | Not demonstrated | 636,919 PLN/QALY  (147,921 EUR/QALY) |  |  |
|  |  | FCR | Indirect (naïve) | Uncertain | Not performed | Not applicable | Not applicable |  |  |
| Romania | Acalabrutinib | Ibrutinib | Nor applicable | Not applicable (HTA did not assess clinical superiority) | CMA | Not applicable | Not applicable | 6 months to 1 year | Not implemented |
| Slovakia | Ibrutinib | TBC | TBC | Not demonstrated | CMA | Not applicable | Not applicable | <6 months | Implemented (details not publicly available) |
|  | Venetoclax | RCD | Direct | Demonstrated | CUA | Demonstrated | Not publicly available | <6 months | Implemented (details not publicly available) |
|  | Venetoclax  +rituximab | Ibrutinib | Indirect | Demonstrated | CEA | Demonstrated | Not publicly available | <6 months | Implemented (details not publicly available) |
| Ukraine | Ibrutinib | HDMP+rituximab | Indirect (naïve) | Uncertain | CEA | Uncertain | Not publicly available | Not reimbursed | Not implemented |
|  | Venetoclax  +rituximab | BR | Direct | Demonstrated | CEA | Not demonstrated | 104,590.63 EUR/LY | Not yet reimbursed | Managed entry agreement recommended |
|  |  |  |  |  | CUA | Not demonstrated | 55,579.37 EUR/QALY |  |  |

BFR, bendamustine + fludarabine + rituximab; BO, bendamustine + ofatumumab; BR, bendamustine + rituximab; BSC, best supportive care; FCR, fludarabine + cyclophosphamide + rituximab; RCD, rituximab + cyclophosphamide + dexamethasone; SOC, standard of care
^a^ Standard of care was defined as BR, HDMP + rituximab, RCD, FCR-lite or chlorambucil+rituximab.

# Supplementary Figures

Supplementary Figure 1. Kaplan–Meier curve: time to reimbursement of R/R CLL drugs

CLL, chronic lymphocytic leukemia; EMA, European Medicines Agency; KM, Kaplan-Meier; NE, not estimable; NR, not reached; R/R, relapsed/refractory. Note: Kaplan–Meier curves reflect the time to national reimbursement, with data censored as of 01.01.2025 (cut-off date). Countries without a reimbursement decision by that date were treated as censored observations. This type of analysis illustrates variability in time to access and allows inclusion of incomplete cases; however, it is limited by the small number of observations and should be interpreted with caution. Median times to reimbursement derived from the Kaplan–Meier estimates tend to be longer than medians calculated only from reimbursed countries, as they account for ongoing delays in access. Median time [95% CI] to reimbursement (months, KM estimates): acalabrutinib: 30.4 [0, 64.0], ibrutinib: 29.3 [21.5, 37.1], zanubrutinib: NR [NE, NE], venetoclax: 37.3 [19.6, 55.0], idelalisib: NR [NE, NE]. Duvelisib was excluded from time-to-event analysis as it was not reimbursed in any of the included countries.
